# Beyond poverty, tungiasis is associated with family characteristics and parenting behavior: a case control study in Kenya

**DOI:** 10.1186/s12889-026-26231-9

**Published:** 2026-01-13

**Authors:** Lynne Elson, Abneel K. Matharu, Berrick Otieno, Naomi Riithi, Paul Ouma, Francis Mutebi, Charles Waiswa, Hermann Feldmeier, Amina Abubakar, Jürgen Krücken, Ulrike Fillinger

**Affiliations:** 1 KEMRI-Wellcome Trust Research Programme, Kilifi, Kenya; 2 Centre for Tropical Medicine and Global Health, Nuffield Department of Medicine, University of Oxford, Oxford, UK; 3 International Centre of Insect Physiology and Ecology (ICIPE), Human Health Theme, Nairobi, Kenya; 4 Institute for Human Development, Aga Khan University, Nairobi, Kenya; 5 College of Veterinary Medicine, Animal Resources and Biosecurity, Makerere University, Kampala, Uganda; 6 Institute of Microbiology, Infectious Diseases and Immunology, Charité University Medicine, Berlin, Germany; 7 Institute for Parasitology and Tropical Veterinary Medicine, Freie Universität Berlin, Berlin, Germany; 8 Veterinary Centre for Resistance Research, Freie Universität Berlin, Berlin, Germany

**Keywords:** Tungiasis, NTDs, Risk factors, Mental health, Parenting style

## Abstract

**Background:**

Tungiasis is a neglected tropical skin disease caused by the sand flea *Tunga penetrans*. Female fleas burrow into the skin, typically of the feet, producing inflammation, pain, and itching. Although poverty is a major risk factor, not all households or children in the lowest economic bracket are affected, and boys appear disproportionately infected. This study investigated environmental and behavioral characteristics of households and children to explain these variations.

**Methods:**

A total of 3,871 pupils (equal numbers of boys and girls) aged 8–14 years from 44 primary schools in Kwale and Siaya counties, Kenya, were examined for tungiasis. In each school, infected and uninfected pupils were randomly selected for household observations and caregiver interviews. Overall, 273 cases and 548 controls were enrolled, from whom 198 infected and 199 uninfected pupils were selected for in-depth interviews. Mixed-effects logistic regression was used to identify risk factors at individual and household levels. Separate models were run for Kwale and Siaya due to contextual differences, and for boys and girls to explore sex-specific determinants.

**Results:**

At household level, tungiasis was associated with higher odds in Muslim households in Kwale (aOR 2.44, 95% CI 1.28–4.62) and traditionist households in Siaya (aOR 2.27, 95% CI 1.06–4.86) compared to Christian households. Additional risk factors included having a male caregiver (Kwale: aOR 2.31, 95% CI 1.02–5.23), a child with disabilities (Siaya: aOR 7.19, 95% CI 1.64–31.65), and lack of caregiver involvement in schoolwork (Siaya: aOR 1.90, 95% CI 1.13–3.19). For girls, infection odds were higher if parents rarely attended school meetings (aOR 2.11, 95% CI 1.00–4.44) or when mothers were frequently absent (aOR 2.46, 95% CI 1.07–5.64). Caregiver stress scores were positively associated with infection risk across sexes (aOR 1.03, 95% CI 1.00–1.06).

**Conclusion:**

This study identifies novel risk factors for tungiasis beyond poverty, including caregiver characteristics, psychosocial stress, and parenting practices. Effective control interventions should integrate psychosocial support for caregivers and promote positive parenting alongside traditional One Health prevention and treatment strategies.

**Trial registration:**

not applicable.

**Supplementary Information:**

The online version contains supplementary material available at 10.1186/s12889-026-26231-9.

## Background

Tungiasis is a neglected tropical skin disease caused by penetration of the female sand fleas, *Tunga penetrans*, mostly at the toes and other sites of the feet. Once fully embedded in the skin, the flea imago grows about 2000 times in volume in a period of about seven days as eggs develop in its abdomen [1]. The eggs are expelled from the tip of the abdomen that protrudes from the host’s skin and drop into the environment. If conditions are appropriate, the larvae will hatch from the eggs, feed, and pupate within one to two weeks [2–4]. When the adult fleas emerge, they actively seek a host. The growth of the embedded flea within the skin causes considerable inflammation, which in turn leads to pain, itching, immobility, and loss of nails [5, 6]. Patients with tungiasis are often stigmatized and discriminated against in their communities [7–9]. Infected pupils have higher school absenteeism, lower academic achievement, and lower quality of life [10, 11].

While the global disease burden is unknown, a recent survey in Kenya found that the national prevalence was 1.35% among school pupils aged 8 to 14 years [12], the age group often with the highest prevalence in a community [13]. This approximates to 171,500 infected children aged 8 to 14 years based on population estimates for 2019 [14]. In some regions, such as northeastern Uganda [15] and southern Ethiopia [16], the prevalence is over 50%. It has been suggested for a long time, and recently been confirmed, that the main risk factor for tungiasis is poverty [12] and it underlies most of the other risk factors identified such as sleeping in a house with an unsealed floor [17–19], not wearing closed shoes [16, 19, 20], practicing open defecation [17, 19, 21], using an unimproved water sources [22], and not washing feet frequently or using soap [13, 23, 24]. However, within the lowest socioeconomic group in a community, for whom all these circumstances apply, not everyone is infected, suggesting that other intrinsic and extrinsic factors impact disease risk. This study aimed to identify environmental and behavioral characteristics of households and children that may be responsible for the fact that not all households or children in the lowest economic bracket are infected, by enrolling infected and uninfected households who live in a house with an unsealed earthen floor. We also aimed to identify factors that may explain the higher prevalence reported for boys in almost all previous studies [12, 17, 18] and in the current research project [25].

## Methods

### Study design

The study used a case: control design and involved children aged 8 to 14 years in primary schools, and their households, in Siaya and Kwale counties of Kenya, as reported previously [25]. The study was carried out between February 2020 and April 2021.

### Study area and population

**Fig. 1:**
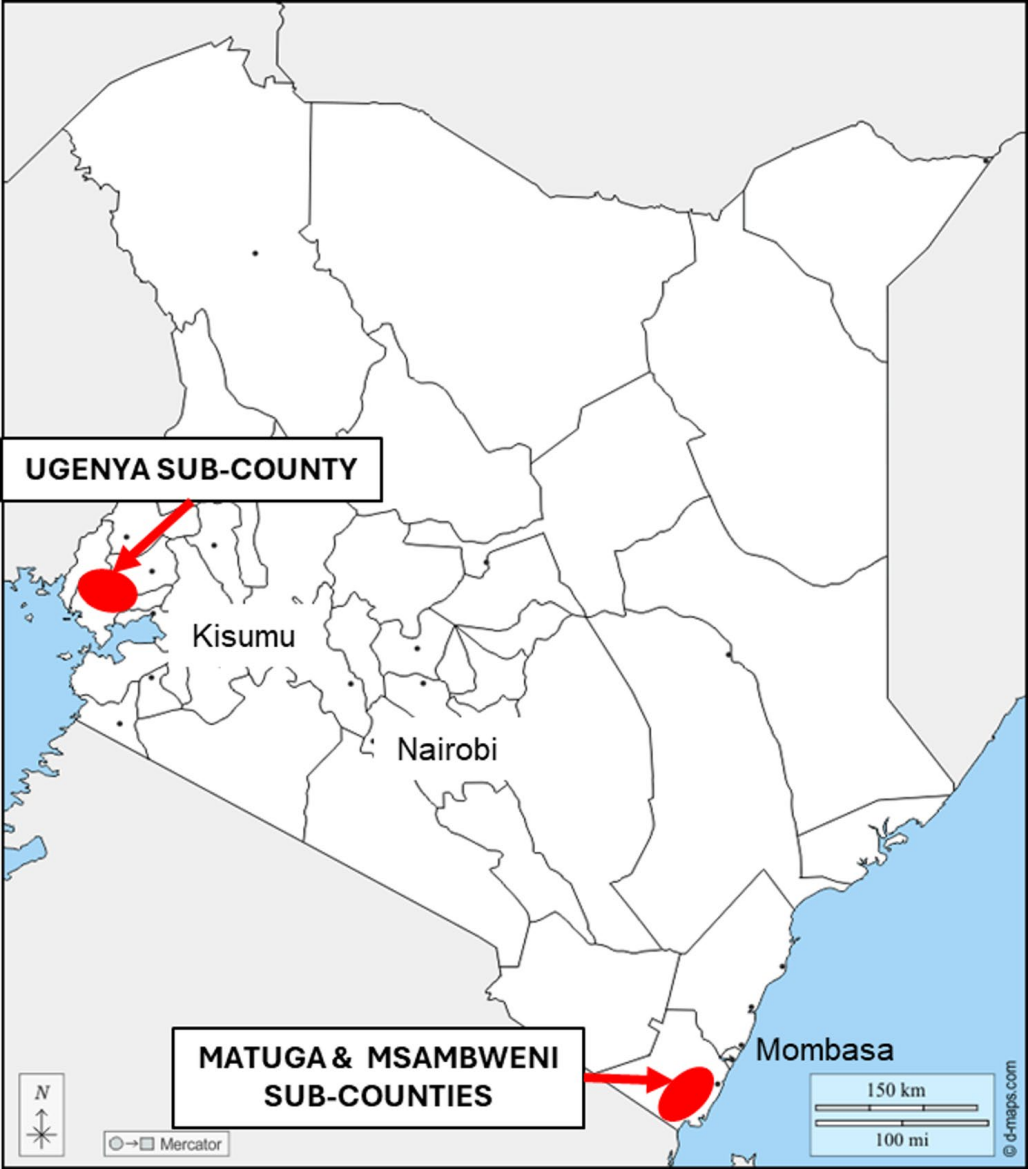
Map of Kenya showing the administrative county boundaries and the two study sites highlighted with a red marker. Base map obtained from https://d-maps.com/carte.php?num_car=236&lang=en

The study was conducted in the sub-counties of Matuga and Msambweni of Kwale county on the southeast coast of Kenya, and in Ugenya sub-county of Siaya county in western Kenya near the border with Uganda (Fig. 1). These areas were chosen as they were highlighted by the Ministry of Health to have a high prevalence of tungiasis [26]. In addition, they have similar climatic conditions. Sugar cane growing, combined with subsistence farming, are the major sources of income for the inhabitants in both regions, but the populations of the two areas differ by ethnicity and culture.

### Sample size

For the case: control study of households, we based our sample size estimate on a previous household risk factor study in Kenya, where cases had a two-fold higher odds than controls of not washing their feet twice a day and 20% of the controls did not wash their feet twice a day [13]. Thus, we estimated for an unmatched case-control study with a ratio of two controls to one case, assuming that 20% of the controls are exposed, and a least extreme odds ratio of 2 to be detected, we would require 126 cases and 252 controls to have 80% power at a 95% confidence level (based on OpenEpi Version 3). However, based on the lack of sufficient baseline data for the study areas, and since we planned to implement a multivariable analysis to identify risk factors, we increased the sample size by 30% guided by rules of thumb [27] and practical considerations. Consequently, our target sample size was at least 160 case households and 320 control households per county, hence a total of 320 cases and 640 controls.

### Sampling procedure

Within Kwale and Siaya counties, sub-counties that were known by the county Departments of Health to have a high tungiasis burden were selected. Lists of all existing public primary schools in the sub-counties were provided by the county education department, and schools were randomly selected using a paper lottery approach. In each school, 51 boys and 51 girls between the ages of 8 and 14 years (the age with the highest prevalence and intensity of tungiasis [22]) were quasi-randomly selected by lining the pupils up into three age groups: 8 and 9 years, 10 and 11 years, and 12 to 14 years), and by sex within each age group. Every n^th^ (depending on the total number in the group) pupil was then selected in each sex and age group until 17 from each was reached, to a total of 102.

The feet of selected pupils were washed and dried and systematically examined for tungiasis, and a short questionnaire was administered to each of the pupils, asking about the floor in the room in which they sleep (sealed concrete floor, or natural soil floor) and whether other people in the family were infected with tungiasis. Pupils sleeping on a sealed concrete floor were not eligible to be included in the case control household survey, as the type of floor has been shown in many studies as a strong risk factor for disease, with sealed floors being highly protective [8, 25, 26]. Our aim was to explore risk factors beyond the floor; hence, cases and controls were drawn from pupils who reported sleeping in a room with an earthen floor. Of the eligible infected pupils in a school, a maximum of ten were randomly selected (paper lottery method) for household surveys. If only ten, or less than ten, were identified in a school, all were selected. Of all eligible uninfected pupils who reported no other family member was infected, 20 were randomly selected from each school, plus 10 reserves, using the paper lottery method. These selected pupils are referred to as the “index child” throughout the manuscript.

While schools were closed due to COVID-19 from March to December 2020, the survey strategy was adapted to recruit cases and controls through a community-based survey in the catchment areas of 11 schools already selected randomly before COVID-19 closures. Details of the procedures can be found in Elson et al. [25]. Out of all the pupils selected for household-based surveys per school, a further random selection of pupils was conducted from among the 10 infected cases and 20 uninfected controls to obtain 6 infected and 6 uninfected for pupil interviews.

### Study procedures

The location of the family homesteads of the selected index children were identified with the support of community health promoters. On arrival at the household, the study and proposed household-based procedures were explained to the household members, and informed consent was sought from the household head, or their representative, usually the main caregiver. Adults (18 years and above) who were to be interviewed provided written consent for the household survey, and for the mental health questionnaire, if applicable, as well as for consent to interview the index child. Additionally, informed assent was sought from children aged 12 to 14 before their interview. In all selected households, the material of the house floor and the household tungiasis infection status were confirmed prior to enrollment. Index children were either interviewed at school or in their homes in privacy to ensure confidentiality. The main caregiver of the family was interviewed at the home, in a location which afforded privacy, after which observations of the homestead structures were made in the presence of the respondent. The interviews and observations were conducted using structured questionnaires, which had been translated into Kiswahili for Kwale and Dholuo for Siaya, and were pre-tested in the two areas. Interviews were conducted by experienced field enumerators who received extensive training in interview techniques and electronic data collection prior to commencement of the study.

### Explanatory variables

In this study, we aimed to explore various factors related to a household that were considered as potentially relevant risk factors for tungiasis. These included questions and observations regarding family demographics, index child school attendance, access to water and sanitation, hygiene behavior, ownership of domestic animals, and household assets to generate a socio-economic status variable. The instrument also collected information on whether they were part of a larger homestead with other households, what building structures belonged to the household, their construction materials, state of repair, and cleanliness, and who slept in each building and room. For this study, the term “household” refers to adults and children who normally eat together but may sleep in more than one building unit in a cluster.

To explore the association of tungiasis with child neglect by caregivers, we included questions relating to parenting behaviors such as discipline methods, time spent with the index child, whether they hug the child, know the child’s friends and their parents, how often they attended a school meeting and talked to the teachers, checked on homework being done, and reading to the child. As a possible cause of neglect, we also asked about family disability, other illnesses, and the caregiver’s mental health. Variables for caregiver depression and parental stress were obtained from another part of this study, which has been published previously [28]. Depression was assessed using the Patient Health Questionnaire-9 (PHQ-9) [29], a self-report in which they rated the frequency of nine symptoms in the past two weeks. Stress was assessed using the Parents’ Stress Scores [30] in which they rate how often they experience stress related to 18 aspects of parenting. The household and pupil questionnaires are included in Additional file 1 and 2. A socioeconomic status (SES) variable was generated for each household using polychoric principal component analysis incorporating ownership of tv, radio, mobile phone, bicycle, motorcycle, solar, and livestock as detailed in section S1 of Additional file 3.

### Data management and analysis

Data were collected and managed using REDCap electronic data capture tools through handheld devices [31]. Data storage was hosted at the International Centre of Insect Physiology and Ecology (ICIPE) in Nairobi, Kenya. All analyses were conducted in Stata/BE 18 (Stata Corp LLC, College Station, TX, USA). Generalized mixed effect logistic models were used to assess associations of variables with household or individual pupil infection. These explanatory variables were collected during the pupil and caregiver interviews and the observations of the homestead. Some variables from the caregiver interview were also included in the pupil data set including the age and sex of head of household and caregiver, the family socio-economic status, whether there were birth complications, the amount of time a caregiver spent with the child and whether they hugged the child. Some variables were not included in the analysis since the number of observations in at least one of the categories was less than 10% of the total number of observations.

The dependent variable for household risk factors was the household infection status (case household vs. control household). The household was classified as a case if at least the index child was infected. In control households, the index child was not infected and there were no other cases in the household, that was confirmed during the visit. To identify factors that may be associated with a household having at least one tungiasis case, multilevel mixed effects logistic models were used with an exchangeable correlation matrix. To reduce any bias caused by the clustered study design, the unique school identifier was included as a random effect. The household models were run separately for Kwale and Siaya due to the numerous differences between them. The dependent variable for pupil risk factors was the infection status of the interviewed index child, either infected or not, and models were run for girls and boys combined and separated. A total of five models were run: Kwale households, Siaya households, children, boys, and girls.

Initially, bivariable analyses were run for each independent variable, and then any variable with a p-value less than 0.2 was included in multivariable analysis. Backward elimination was used to develop the final model using Akaike information criteria (AIC) to compare the goodness of fit of the models. Wald tests were used to confirm the role of variables in the final models. Bivariable outcomes are presented as odds ratios (OR) and multivariable outcomes as adjusted odds ratios (aOR) with 95% confidence intervals and p-values.

## Results

### Study population

**Fig. 2:**
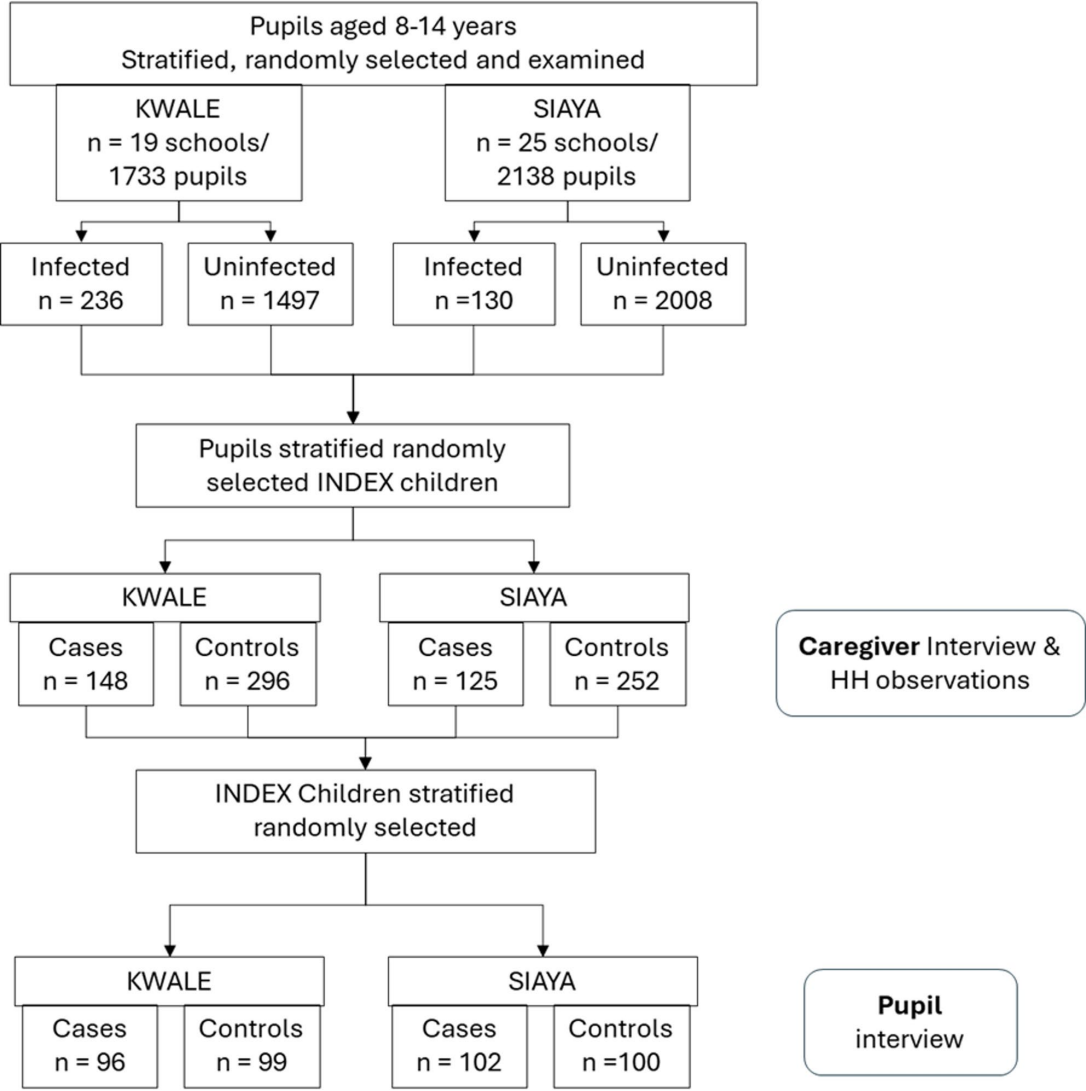
Study flow chart illustrating the selection of participating pupils and households (HH)

A total of 44 schools (Kwale 19, Siaya 25) were enrolled, and 3,871 pupils were examined for tungiasis, 1733 in Kwale and 2138 in Siaya. The total number of pupils was less than the expected 4,488 since some schools were small and had fewer than 102 pupils in the target age group present on the day of the examinations. Of the examined pupils, 236 (13.6%) in Kwale, and 130 (6.1%) in Siaya, were found to be infected. Details on infection prevalence and disease severity have been published elsewhere [25]. In Kwale, 148 cases and 296 controls were selected as index children for household interviews and observations. In Siaya, 125 cases and 252 controls were selected (Fig. 2). A further round of sampling was conducted among the index children to select those for individual pupil interviews: 96 cases and 99 controls in Kwale, 102 cases and 100 controls in Siaya.

### Household characteristics

In both counties, most households selected were headed by a male (Kwale 80.1%, Siaya 73.7%) with a median age of 45 years (IQR 38–56) while most primary caregivers were female (Kwale 92.6%, Siaya 91.2%) with a median age of 36 years (IQR 30−42) in Kwale and 39 years (IQR 32–48) in Siaya (Table 1).

There were significant differences between the households in Kwale and Siaya. The socioeconomic status of the households was higher in Siaya than Kwale and probably as a consequence a higher percentage of households in Siaya than in Kwale had a separate kitchen (70.8% v. 51.8%), a built stone bathroom (12.2% v. 5.4%), and had a main house roof made with iron sheets (91.2% v. 36.7), while fewer households used open defecation (8.2% v. 40.5%), (Table 1). Households in Siaya were mostly Christian (86.2%) while those in Kwale were mostly Muslim (79.3%). Of the 49 households that reported following a traditional religion, 46 (93.9%) were in Siaya. Further household variables are included in Supplementary Table S2 in Additional file 3.

**Table 1:** Household characteristics by county

Variable	Category	Kwale	Siaya	Total	*P*-value^a^
		*N* (%)^b^	*N* (%)	*N*	
All		444	377	821	
HHH^c^ sex	Male	356 (80.2)	278 (73.7)	634	0.028
	Female	88 (19.8)	99 (26.3)	187	
Caregiver sex	Male	33 (7.4)	33 (8.8)	66	0.488
	Female	411 (92.6)	344 (91.2)	755	
Household religion	Muslim	352 (79.3)	4 (1.1)	356	< 0.001
	Christian	89 (20.0)	325 (86.2)	414	
	Traditionist	2 (0.5)	42 (11.1)	44	
Marital status of caregiver	Married	347 (78.2)	287 (76.1)	634	< 0.001
	Widow	44 (9.9)	75 (19.9)	119	
	Single	52 (11.7)	9 (2.4)	61	
Own land where house is	Yes	403 (90.8)	355 (94.2)	758	0.068
	No	41 (9.2)	22 (5.8)	63	
Own farmland	No	273 (61.5)	107 (28.4)	380	< 0.001
	Yes	171 (38.5)	269 (71.4)	440	
Part of a homestead	No	259 (58.3)	250 (66.3)	509	0.017
	Yes	185 (41.7)	126 (33.4)	311	
Have separate kitchen building	No	214 (48.2)	110 (29.2)	324	< 0.001
	Yes	230 (51.8)	267 (70.8)	497	
Have a separate hut for teenagers	No	339 (23.6)	270 (71.6)	609	0.123
	Yes	105 (23.6)	107 (28.4)	212	
Bathing place	Built stone	24 (5.4)	46 (12.2)	70	0.001
	other	420 (94.6)	331 (87.8)	751	
Toilet	Open defecation	180 (40.5)	31 (8.2)	211	< 0.001
	Latrine	264 (59.5)	346 (91.8)	610	
Number adults	0–1	38 (8.6)	86 (22.8)	124	< 0.001
	2–4	368 (82.9)	280 (74.3)	648	
	> 4	38 (8.6)	11 (2.9)	49	
Number under 5-year-olds	0	146 (32.9)	161 (42.7)	307	< 0.001
	1–2	276 (62.2)	183 (48.5)	459	
	> 2	22 (5.0)	33 (8.8)	55	
Number 6-17-year-olds	1–2	240 (54.1)	251 (66.6)	491	< 0.001
	> 3	204 (45.9)	126 (33.4)	330	
Walls of main house	Stone/ brick	48 (10.8)	16 (4.2)	64	0.001
	Mud/mixed	396 (89.2)	361 (95.8)	757	
Roof of main house	Iron sheet	163 (36.7)	344 (91.2)	507	< 0.001
	Natural	281 (63.3)	32 (8.5)	313	
Number of sleeping rooms in main house	1	83 (18.7)	227 (60.2)	310	< 0.001
	2	213 (48.0)	143 (37.9)	356	
	> 2	148 (33.3)	7 (1.9)	155	
Age (median (IQR)	HHH	45 (38−56)	46 (39−58)	46 (39−57)	0.777^d^
	Caregiver	36 (30−42)	39 (32−48)	37 (31−45)	< 0.001
Socio-economic status Mean (sd)		0.56 (0.36)	0.71 (0.35)		< 0.001^e^

^a^ p value from Chi^2^ test Kwale vs. Siaya, ^b^ number and percent of all observations in county, ^c^ head of household, ^d^ p value from Mann-Whitney test for Kwale vs. Siaya, ^e^ p-value from T-test for Kwale vs. Siaya

### Household risk factors

Since households in the two counties were different in many of their characteristics, risk factor analyses were conducted separately. Despite exploring many factors related to the home environment, covering family structure, physical infrastructure, socio-economic status, socio-behavioral factors, hygiene, and sanitation factors in the bivariable analysis, few were found to be associated with having tungiasis in the household, both in Kwale and Siaya (Supplementary Table S3 in Additional file 3). While several factors did not have p-values less than 0.05, they were retained in the models because their removal caused an increase in the AIC, and LR-tests suggested the factor played some role in the model. The final multivariable models for the two counties were different from each other.

### Kwale

In the multivariable model for Kwale (Table 2), when adjusting for pupil age and sex, which were both associated with household infection, several new factors were identified. The few (6.9%) households whose main caregiver was male had twice the odds of infection than the majority who had a female caregiver (aOR 2.31, 95% CI 1.02−5.23, *p* = 0.045). In Kwale, 79% of households reported practicing Islam, and they had twice the odds of being infected than the 20% who practiced Christianity (aOR 2.4, 95% CI 1.28−4.62, *p* = 0.006). The number of meals a household ate the previous day was negatively associated with tungiasis infection, that is, the more meals eaten in a day the lower the odds (aOR 0.48, 95% CI 0.28−0.84, *p* = 0.010).

Other new variables associated with tungiasis in Kwale related to the house and sleeping room of the index child. The 30.2% of households whose main house was not in a good state of repair had twice the odds of infection than those that were good (aOR 2.10, 95% CI 1.28−3.44, *p* = 0.003). The odds of infection for a household was highest when boys and girls slept in the same room (aOR 1.85, 95% CI 1.01−3.36, *p* = 0.045).

Factors that have been previously published for tungiasis and that were also found to be associated with higher odds of tungiasis in Kwale included using an unimproved water source, the number of adults in the household, and not sleeping on a raised bed, but on the floor (Table 2).

**Table 2:** Kwale household risk factors for tungiasis status. Mixed effects multivariable logistic regression with school as random effect (*n* = 439). Bivariable results in supplementary table S3 in additional file 3

		KWALE					
Variables	Categories	*N*^a^ (%)	aOR^b^	95% CI^c^		*P* ^d^	
Pupil age			0.87	0.77	0.98	0.026	
Pupil sex	Female		1				
	Male		2.66	1.51	4.71	0.001	
Caregiver sex	Female	389 (93.1)	1				
	Male	29 (6.9)	2.31	1.02	5.23	0.045	
Family religion	Christian	84 (20.1)	1				
	Muslim	331 (79.2)	2.44	1.28	4.62	0.006	
	Traditionist	3 (0.7)	-				
Water source	Improved	376 (89.9)	1				
	Not improved	42 (10.1)	2.38	1.15	4.94	0.019	
Number of meals eaten the previous day			0.48	0.28	0.84	0.010	
State of repair of main house	Good	310 (69.8)	1				
	Not good	134 (30.2)	2.10	1.28	3.44	0.003	
Number of adults in HH^e^			2.25	1.28	3.95	0.005	
Index child sleeping place	With adults	159 (38.0)	1				
	Mixed kids	96 (22.9)	1.85	1.01	3.36	0.045	
	Boys only room	77 (18.4)	1.22	0.63	2.36	0.564	
	Girls only room	67 (16.0)	0.78	0.33	1.85	0.574	
	Grandparents room	12 (2.9)	0.65	0.16	2.69	0.548	
	Lounge	7 (1.7)	0.52	0.09	3.10	0.472	
	Kitchen	0					
	Separate hut	0					
	Neighbor’s hut	0					
Index child sleeps on a raised structure	Yes	343 (82.1)	1				
	No	75 (17.9)	2.66	1.51	4.67	0.001	

^a^Number of households; ^b^adjusted odds ratio; ^c^confidence interval; ^d^p value; ^e^ household

### Siaya

In the multivariable model for Siaya, when adjusting for index child age and sex, which were both associated with the household infection status, some new factors were identified. Only three families practiced Islam, but 46 families practiced a traditional religion, and they had twice the odds of infection than the majority (86%) of families who practiced Christianity (aOR 2.27, 95% CI 1.06−4.86, *p* = 0.018) (Table 3). While tungiasis is known to cause physical disability, we have explored whether children who already have a physical or mental disability have a higher odds of infection. Only 11 index children in Siaya were identified as having a disability, which caused a wide 95% confidence interval, but their households had seven times higher odds of being infected (aOR 7.19, 95% CI 1.64−31.65, *p* = 0.009). Disability was not diagnosed formally by a clinician, but what was obvious to the enumerators and confirmed with the local community health worker or caregiver.

Despite exploring multiple factors related to parenting behavior as proxy indicators of child neglect, only one was retained in the model. Households where the parent reported they had not checked their child’s homework in the previous week had twice the odds of being infected than those who did (aOR 1.90, 95% CI 1.13−3.19, *p* = 0.015).

As in Kwale, tungiasis was associated with the room the index child slept in. The odds of infection were higher if the index child slept with their grandparents (aOR 3.38, 95% CI 0.97−11.72, *p* = 0.055) or in the lounge (aOR 2.53, 95% CI 1.01−6.37, *p* = 0.048). For the 14% of index children who slept in a room that was not swept and tidy, the household had nearly four times higher odds of infection than those whose room was swept and tidy (aOR 3.54, 95% CI 1.76–7.15, *p* < 0.001). In addition, a household had higher odds if the index child slept on rags or a mat rather than a mattress (aOR 2.26, 95% CI 1.34–3.80, *p* = 0.002).

**Table 3:** Siaya household risk factors for tungiasis status. Mixed effects multivariable logistic regression with school as random effect (*n* = 366). Bivariable results in supplementary table S3 in additional file 3

		SIAYA				
Variables	Categories	*N* ^a^	aOR^b^	95% CI^c^		*P* ^d^
Pupil age			0.85	0.74	0.97	0.018
Pupil sex	Female	149 (42.7)	1			
	Male	200 (57.3)	3.87	2.20	6.79	< 0.001
Family religion	Christian	302 (86.0)	1			
	Muslim	3 (0.9)	1.29	0.14	12.01	0.823
	Traditionist	46 (13.1)	2.27	1.06	4.86	0.035
Index child had a disability	No	340 (96.9)	1			
	Yes	11 (3.1)	7.19	1.64	31.65	0.009
Caregiver checked index child homework in last week	Yes	216 (62.1)	1			
	No	132 (37.9)	1.90	1.13	3.19	0.015
Index child sleeping place	In adults’ room	45 (12.8)	1			
	Mixed kids’ room	26 (7.4)	1.72	0.51	5.81	0.383
	Boys only room	52 (14.8)	1.98	0.72	5.41	0.185
	Girls only room	2 (0.6)	3.64	0.20	67.91	0.387
	Grandparents’ room	22 (6.3)	3.38	0.97	11.72	0.055
	Lounge	98 (27.9)	2.53	1.01	6.37	0.048
	Kitchen	99 (28.2)	1.24	0.48	3.26	0.656
	Separate hut	5 (1.4)	4.95	0.54	45.16	0.156
	Neighbor’s hut	2 (0.6)	1.12	0.05	23.68	0.94
Surface index child sleept on	Mattress	200 (57.0)	1			
	Rags, mats etc.	151 (43.0)	2.26	1.34	3.80	0.002
Sanitation of index child’s room	Swept & tidy	299 (85.7)	1			
	Not swept & tidy	50 (14.3)	3.54	1.76	7.15	< 0.001

^a^ Number of households; ^b^ adjusted odds ratio; ^c^ confidence interval; ^d^ p value

### Index child risk factors

Of the 395 pupils selected for the in-depth interview, by design, there were almost equal numbers from Kwale (193) and Siaya (202), 57% (227) were male, and their mean age was 10.8 years (standard deviation 2.0). The distribution of girls and boys by all variables is shown in Table 4 and Supplementary Table S4 in Additional file 3, which shows a significant difference between the sexes in their infection status and in the frequency which they washed their feet.

**Table 4:** Demographic characteristics of the pupils by sex

Variable	Category	All *N*^a^ (%)	Female *N* (% of female)	Male *N* (% of male)	*p*-value
All		395	168	227	
Tungiasis status	Uninfected	197	111 (66.1)	86 (37.9)	<0.001
	Infected	198	57 (33.9)	141 (62.1)	
Region	Kwale	193	88 (52.4)	105 (46.3)	0.229
	Siaya	202	80 (47.6)	122 (53.7)	
Shoes worn	Closed	95	43 (25.6)	52 (22.9)	0.537
	Other	300	125 (74.4)	175 (77.1)	
Uniform worn	Complete	70	32 (19.1)	38 (16.7)	0.553
	Other	325	136 (81.0)	189 (83.3)	
HHH^b^ sex	Female	87	35 (21.7)	52 (23.0)	0.768
	Male	300	126 (78.3)	174 (58.0)	
Caregiver sex	Female	354	151 (93.8)	203 (89.8)	0.169
	Male	33	10 (6.2)	23 (10.2)	
Child feared a family member	No	264	104 (61.9)	160 (70.8)	0.063
	Yes	130	64 (38.1)	66 (29.2)	
Parents knew child’s friends	No	42	14 (8.3)	28 (12.3)	0.202
	Yes	353	154 (91.7)	199 (87.7)	
Parents knew child’s friends’ parents	No	62	24 (14.3)	38 (16.7)	0.507
	Yes	333	144 (85.7)	189 (83.3)	
Parents attend school meetings	No	189	82 (49.1)	107 (47.1)	0.700
	Yes	205	85 (50.9)	120 (52.9)	
Bed child slept on	Raised bed	208	95 (56.6)	113 (49.8)	0.183
	On floor	187	73 (43.5)	114 (50.2)	
Frequency child washed feet	Twice a day	293	135 (81.8)	158 (69.9)	0.007
	Less often	98	30 (18.2)	68 (30.1)	
Soap-use for feet washing	Always	150	67 (39.9)	83 (36.7)	0.524
	Less	244	101 (60.1)	143 (63.3)	
Toilet used	Open defecation	72	33 (19.6)	39 (17.2)	0.531
	Latrine	323	135 (80.4)	188 (82.8)	
Caregiver depression	No	253	110 (65.5)	143 (63.0)	0.611
	Yes	142	58 (34.5)	84 (37.0)	

^a^ Number of participants, ^b^ head of household

When models were run for all pupils together, age and sex of the pupil were significantly associated with infection, with the odds of infection decreasing with each year of age and boys having three times higher odds of infection than girls (aOR 3.58, 95% CI 2.28−5.71, *p* < 0.001, Table 5). Since we aimed to identify factors that could be associated with the higher odds for boys, we also ran the bivariable and multivariable logistic regression models separately for girls and boys. The bivariable results are presented in Supplementary Table S5 in Additional file 3, while the final multivariable models are presented together in Table 5.

Of all the many factors assessed, very few were significantly associated with infection, particularly for boys. For several factors, the p-value was not less than 0.05, but if they were removed from the models, the AIC increased considerably. If one of the 95% confidence limits was close to 1.00, they were retained in the models since this may be indicative of a relationship that deserves further investigation.

Several new factors were identified that had not been published previously. Since caregiver stress and depression had previously been linked to child neglect [32, 33] and child neglect has been linked to poor health outcomes among children, we investigated whether these factors might be associated with tungiasis infection among children. When the caregivers of the pupils were assessed using the parental stress score and PHQ-9, it was established that 35.9% (142) of the caregivers were depressed [28]. Although both caregiver depression and parental stress were significantly associated with a pupil’s infection status in the bivariable models when the sexes were analyzed together, only parental stress was retained in the combined final multivariable model, with the odds of infection increasing for each unit increase in the stress score (aOR 1.03, 95% CI 1.00−1.06, *p* = 0.025). When the models were run separately for boys and girls, caregiver depression was associated with infection for boys only (aOR 1.75, 95% CI 0.96−3.21, *p* = 0.070) while for girls, the parental stress score was positively associated with their infection (aOR 1.06, 95% CI 1.01−1.11, *p* = 0.030).

Proxy indicators for child neglect were assessed, including parents being away a lot, parents attending school events, checking on homework, reading to a child, knowing the child’s friends, and their parents. However, only the variable for parents attending school meetings was associated with tungiasis for all pupils, and this only held true for girls when assessed separately. The 205 (52.0%) pupils whose parents did not attend school meetings often had nearly twice the odds of infection than pupils whose parents attended often (aOR 1.79, 95% CI 1.13−2.83, *p* = 0.013). When assessed separately, this was only significant for girls, who had twice the odds of infection than those girls whose parents did attend school meetings often (aOR 2.11, 95% CI 1.00−4.44, *p* = 0.049). Lastly, the 47 (29%) girls who said their mother was away from home a lot had twice the odds of infection than girls whose mother was not (aOR 2.46, 95% CI 1.07−5.64, *p* = 0.033). There was no association with father’s absence, nor for either parents’ absence for boys.

Infection among boys was associated with whether they feared a family member. Although not significant, there was an indication that the 66 (29%) boys who said they feared someone had nearly twice the odds of infection than boys who said they did not fear anyone in the family (aOR 1.68, 95% CI 0.88−3.22, *p* = 0.117). Among the boys who said they feared a family member, 23% feared their father, 17% their brother, and 12% their uncle (Supplementary Table S4 in Additional file 3). Although a higher percentage of girls (39%) said they feared a family member, particularly their father, this was not associated with their tungiasis status. Lastly, the 168 (81%) girls who were not wearing a complete uniform at the time of the interview had six times higher odds of infection than girls who did have complete uniforms (aOR 5.89, 95% CI 1.58−22.04, *p* = 0.008).

Factors that have been published previously but also associated in the current study included not wearing closed shoes, sleeping on a raised bed, and open defecation (Table 5).

**Table 5:** Risk factors for tungiasis of index children combined, and for girls, and boys separately: multivariable logistic regression using school as random effect

		All pupils (*n* = 366)^a^					GIRLS (*n* = 163)^a^						BOYS (*n* = 224)^a^				
Variables	categories	N (%)^b^	aOR^c^	95% CI^d^		P^e^	N	aOR	95% CI		P	N		aOR	95% CI		p
Sex	Female	168 (42.5)	1														
	Male	227 (57.5)	3.58	2.25	5.71	< 0.001											
Pupil age			0.81	0.71	0.92	0.001		0.78	0.65	0.95	0.012			0.86	0.74	1.01	0.062
Shoes worn	Closed	95 (24.1)	1				43	1				52					
	Other	300 (75.9)	2.01	1.15	3.50	0.014	125	2.67	1.06	6.72	0.037	175					
Uniform worn	Complete	70 (17.7)					32	1				38					
	Other	325 (82.3)					136	5.73	1.55	21.28	0.009	189					
Mother away a lot	No	174 (70.1)					119	1				155					
	Yes	117 (29.9)					47	2.46	1.07	5.64	0.033	70					
Parents attend school meetings	Often	189 (48.0)	1				82	1				107					
	Not often	205 (52.0)	1.79	1.13	2.83	0.013	85	2.11	1.00	4.44	0.049	120					
Child feared a family member	No	264 (67.0)					104					160		1			
	Yes	130 (33.0)					64					66		1.68	0.88	3.22	0.117
Bed child slept on	Raised bed	207 (52.7)	1				95					113		1			
	On floor	190 (47.3)	1.65	1.04	2.61	0.034	73					114		1.80	1.02	3.18	0.043
Caregiver depressed ^f^	No	255 (64.1)					110					143		1			
	Yes	142 (35.9)					58					84		1.75	0.96	3.21	0.070
Parental stress score ^g^			1.03	1.00	1.06	0.025		1.06	1.01	1.11	0.03						
Toilet used	Latrine	325 (81.8)	1														
	Open defecation	72 (18.1)	1.93	1.06	3.51	0.032											

^a^ Number of observations in the final model, ^b^ number of pupils, ^c^ adjusted odds ratio, ^d^ confidence interval, ^e^ p value, ^f^ diagnosis from PHQ-9 assessment published elsewhere [28], ^g^ assessed and published elsewhere [28]

## Discussion

In this study, we set out to identify factors that may be associated with tungiasis beyond what had already been published, and beyond factors that are related to poverty, since not all households that are in the lowest socio-economic bracket are infected. To do this, we enrolled pupils and their households in areas known to have a high number of cases, and only from households that lived in houses with an earthen floor, expecting this to enable us to focus on the lowest socioeconomic group. Although new factors were identified, the underlying cause of some of them is likely extreme poverty, revealing that multiple economic groups have earthen floors.

The new risk factors for households fell into three main categories relating to either household infrastructure, parenting, or characteristics of the index children, which were different between Kwale and Siaya, and between girls and boys. For the first time, we report an association of tungiasis in a household with the religion they practice; higher odds were seen with Islam in Kwale and traditional religion in Siaya, relative to Christianity. Also new in this study, household infection was associated with a male caregiver in Kwale and a child having a disability in Siaya. Family sleeping arrangements also seem to play a role in both counties; higher odds for families where children sleep in their grandparents’ room or in the lounge in Siaya, while in Kwale, higher odds were seen for households where boys and girls sleep in the same room. In Siaya, households where the index child’s room was not swept and tidy had higher odds of infection. Only in Siaya were any of the parenting variables associated with household infection. Tungiasis was associated with lower odds of infection in Siaya If the caregiver had checked on the child’s homework in the last week.

To our knowledge, this is the first study to find higher odds of infection associated with the practice of a specific religion. The reasons for the association with practicing a traditional religion are unknown, but perhaps the traditionalist families in Siaya also had other characteristics and behaviors yet unidentified that put them more at risk of infection, such as avoiding health care services, and outreaches which we have alluded to previously [25]. The association with practicing Islam in Kwale is unexpected as the practice of bathing feet multiple times a day before prayers might have been expected to be associated with lower odds of infection, since low frequency of foot washing is associated with tungiasis [13, 24]. However, it appears that in this study (Supporting Information S6 Table), and in others [34], those households that practiced Islam had a lower economic status, so again the higher odds are likely caused by poverty, not practicing the specific religion.

While poor sanitation of a compound has been mentioned as a risk factor for tungiasis [17, 21], this is the first study to assess indoor sanitation and attempt to quantify the cleanliness of a sleeping room. Despite this being a subjective measure, two different teams conducted these assessments in the two counties, and in both counties, the sanitation of the room was associated with tungiasis in the bivariable models (Supporting information Table S2, Additional file 3), but was only retained in the multivariable model for Siaya. The households in Siaya where the sleeping room of the index child was reported to be not swept and tidy had higher odds of infection. Keeping rooms untidy with items scattered around the floor makes it harder to sweep and clean, leaving loose material which shelters the off-host stages and organic material that might be suitable for the larvae to feed on.

In the bivariable models, households with a boys-only room had higher odds, and those with a girls-only room had lower odds of tungiasis. However, adjusting for sex in the multivariable models meant these were no longer significant. This likely reflects the association of tungiasis with sex, boys having higher odds as seen in this study and in many others [17, 18, 35]. The higher odds for those households in Siaya where children and grandparents shared a sleeping room may reflect the higher vulnerability and odds of infection seen for older adults in other studies [13, 17]. The higher odds for the 28% of households where the index child slept in the lounge may reflect the poverty of the household, causing them to only have one sleeping room (60% of households in Siaya). Alternatively, this may reflect a culture in which it is the norm to prioritize having a lounge over a second sleeping room. Note that in Kwale, where the socio-economic status of households was lower than in Siaya, only 19% of households had only one sleeping room.

Another factor that has been thought to increase a person’s vulnerability to infection is disability, but to date, there has been no study to demonstrate this. In the current study and our parallel study in northeastern Uganda [36], we found that households with a child who is disabled, either physically or mentally, had a higher odds of infection. Such individuals spend much of the day sitting on the ground, exposed to adult female fleas. They are also more likely to have poorer levels of hygiene, as they probably rely on others to assist them in maintaining their body hygiene.

Factors that were identified here and have previously been reported in other studies include sleeping on the floor rather than a bed [13, 22], using an unimproved water source [22], and having higher numbers of adults sleeping in a house [13, 22, 37]. All of these are likely related to poverty, but some may also be related to the flea’s biology. If one person sleeping in a room becomes infected, they are likely to contaminate the house floor with eggs. These will develop into parasitic imagines if the conditions are right and infect others sleeping in the room. In addition, in the past, we have demonstrated that free-living adult fleas will jump from an infected host to another host in close proximity [38]. The higher the density of people, the higher the chance a flea can jump from one to another or can find and infect a host after emergence from its pupa. Having a higher density of people also implies more feet moving in and out of the building, which would likely introduce more dirt and organic matter into the rooms, making it harder to keep clean, providing the right conditions for off-host development. While sleeping on the floor and not a raised bed is likely the result of poverty, it puts children at higher risk since they are closer to the host-seeking adult female fleas that emerge from pupae in the soil of the house floor. In Siaya, there was no association with sleeping on a raised bed, but with sleeping on rags or a mat instead of a mattress, either on a raised bed or on the floor. This is also likely a result of extreme poverty.

Most (93%) of the study households had a female caregiver, but our findings indicate that the few households in Kwale, where the caregiver was male, the household had a higher odds of being infected. Male caregivers are likely to be spending most of their time focused on income generation to support the family and will have less time, and perhaps give lower priority to resource use for health and child well-being [39].

The factors that related to pupil characteristics were identified in the pupil interviews, and all were related to extreme poverty. Girls had higher odds of infection if they neither wore closed shoes nor had a complete uniform. Neither of these factors was associated with infection in boys, but they had higher odds of infection if they did not have a raised bed to sleep on and instead slept on the floor.

This study pioneered investigations into caregiver mental health as a risk factor for tungiasis. Although the associations were not very strong, there is a suggestion that caregiver stress and depression are associated with tungiasis in children, which merits further investigation, perhaps through different study designs. A caregiver who is experiencing stress or is depressed is unlikely to give full attention to or care for their children, such as bathing them frequently or making sure they bathe themselves, feeding and clothing them properly, or paying attention to who their friends are or their school achievements [33, 40].

Despite including multiple variables, which we considered to be indicators of parental neglect, only one was associated with household infection when we interviewed caregivers in Siaya, and two were associated with infection when we interviewed pupils, and for girls only. Households where caregivers said they had checked their child’s homework in the last week had lower odds of infection. Girls had higher odds of infection if their parents did not often attend school meetings, and the mother was away a lot. These findings are important as they suggest interventions for tungiasis prevention need to address not only the biology of the parasite, but also caregiver mental health and parenting behavior.

The majority of pupils did not wear any shoes or only wore open sandals on the day of the interview, and they had higher odds than the few wearing closed shoes, corroborating other studies in Kenya and elsewhere [16, 18, 41]. While it is likely that good quality, closed shoes can prevent the penetration of feet by the adult fleas, most transmission seems to occur at home in the sleeping rooms [3, 42] where children do not wear shoes. Therefore, the association with shoe type could reflect protection from transmission in schools that do not have clean, well maintained concrete floors [23, 43]. More likely, it reflects the economic status of the family, even within the economic group defined by having an earthen house floor.

As seen in other studies [10, 24], absenteeism from school was associated with infection. This could be the result either of infected children not being able to walk to school due to the pain experienced or to avoid school due to stigma and shame [7, 11]. Conversely, they may be absent for financial reasons, as was the situation for one third of all pupils in this study. As we have shown previously for this study population, being at home more was associated with a higher prevalence of infection [25].

### Limitations

One limitation of this study may have been its design. We implemented the traditional quantitative method used for identifying risk factors. However, since few factors were found to be significantly associated with tungiasis, and there were weak associations with several sociological and behavioral factors, suggests that future studies should use the qualitative techniques typically used in anthropological and sociological studies. However, so few factors were identified with strong associations when poverty was controlled through participant selection, suggests that the strongest risk factor is extreme poverty.

## Conclusions

We conducted an extensive and in-depth study of factors that could put families and children at risk of tungiasis, but few new risk factors were identified. We also hoped to identify factors that put boys at higher risk than girls, but this study design has not enabled that. Future studies should observe typical behaviors of boys in infected communities, such as their free-time activities, work they do in their families, and their hygiene practices. It is likely that tungiasis is the result of a complex interplay of multiple factors in addition to poverty. We have shown that risk factors vary between locations and between sexes. We did not investigate it here, but it is possible that the factors associated with individual risk may also be different between age groups. Beyond poverty-related factors, disability, caregiver mental health, and parenting style appear to play a role in the occurrence of tungiasis and are deserving of further studies using a more qualitative approach. In the meantime, interventions for tungiasis control will probably be most successful if they address not only treatment and prevention based on the biology of the parasite and poverty but also integrate psychosocial support for caregivers and improve parenting styles. Using a participatory approach involving parents from target communities in the intervention design and implementation is likely to be more effective and sustainable. 

## Supplementary Information

Additional file 1. Household Questionnaire. Microsoft word file containing the questionnaire used to collect data February 2020-April 2021 during interviews of caregivers in Kwale and Siaya counties of Kenya. Contains 10 pages of text, file size 533 Kb

Additional file 2. Pupil Questionnaire. Microsoft word file containing the questionnaire used to collect data February 2020-April 2021 during interviews of pupils aged 8 to 14 years in Kwale and Siaya counties of Kenya. Contains 5 pages of text, file size 263 Kb

Additional file 3. Microsoft word file containing the following sections on 26 pages of text, file size 175 Kb: S1 Polychoric principal component analysis for household socioeconomic status. Table S2. Household population characteristics by county. Table S3. Bivariable mixed effects logistic regression for household risk factors for tungiasis status for Kwale and Siaya. Table S4. Pupil characteristics by sex. Table S5. Bivariable mixed effects models for all pupils and boys and girls separately. Table S6. Factors associated with household socioeconomic status

Additional file 4. Elson et al. Household Dataset.xlsx Description: Microsoft excel workbook containing data and associated codebook for data collected February 2020-April 2021 during interviews of caregivers in Kwale and Siaya counties of Kenya. Contains 243 variables for 697 observations, 621Kb.

Additional file 5. Elson et al. Pupil dataset 28052025.xlsx Description: Microsoft excel workbook containing data and associated codebook for data collected February 2020-April 2021 during interviews of pupils in Kwale and Siaya counties of Kenya. Contains 64 variables for 395 observations, 174 Kb.

## Data Availability

The datasets supporting the conclusions of this article are available in the Additional files associated with this manuscript.
