# Supplementary material for: Beyond poverty, tungiasis is associated with family characteristics and parenting behavior: a case control study in Kenya
Source: BMC Public Health. 2026 Jan 13;26:540. doi: 10.1186/s12889-026-26231-9 (PMC12888424; doi:10.1186/s12889-026-26231-9)
Supplement: Supplementary file 1 — Additional file 1. Household Questionnaire. Microsoft word file containing the questionnaire used to collect data February 2020-April 2021 during interviews of caregivers in Kwale and Siaya counties of Kenya. Contains 10 pages of text, file size 533 Kb [file 12889_2026_26231_MOESM1_ESM.docx]

Household Survey

*Page 1*

ID

Date

Enumerator (interviewer) Initials

Region BU

SI KW


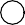

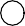

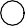


School ID number (as per school list) from where the index child was selected

(e.g 093)

Index child project ID

(e.g. KW_093_001)

Name of index child

(First and family name)

latitude

longitude

Home village

Name of head of household:

Sex of head of household: male

female


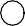

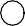


Age of head of household

Name of care giver:

Sex of care giver: Male

Female


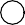

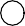


Age of care giver:

Care givers relation to index child? parent grandparent sibling aunt/uncle cousin


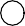

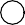

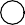

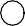

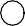

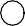


other (specify)

If other relation, what kind?

**Socioeconomic factors**

Do you (your family) own this land on which you have Yes its owned, your house? no its rented,


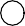

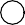

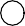

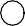


no we are sqatters, no-other reasons.

Other reasons

Does this family own ADDITIONAL land for farming? Yes No

**Select Yes if the family owns any below or NO if not**

Yes No


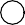

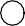


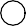

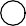

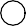

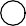
radio tv


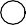

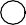

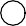

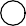
mobile phone bicycle


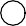

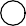

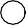

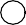
motor cycle solar system


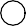

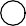
car, tractor or boat

4.Observe and/or ask if there is a local bar Yes

(drinking place) seen from this house? No (observe)


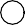

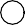


**Water, Sanitation and Hygiene**

What is your PRIMARY (most often used) source of tap in compound


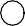

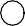

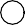

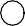

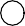

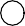

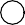


water? shared community tap

own well

shared community well or borehole river or lake or pond

rainwater collection in tank/drums combination - specify

specify water source

Does any of the following apply? (if yes, select,
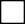
 The household pays for the daily water supply. multiple answers possible)
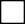
 Some or all of the water needed is brought by a

hired motorbike.


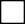
 Some or all of the water needed is brought by a hired donkey cart.


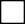
 Some or all of the water needed is fetched by household members from a short distance.


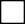
 Some or all of the water needed is fetched by household members from a long distance.


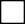
 None of above applies. (read the options )

How many jerry-cans (of large 20 L type) of water do

you use in a day for BATHING (all family members

total)? (Not including water for cooking, drinking or

laundry)

In which location do you bathe most of the time? in river/pond/lake,

with basin of water anywhere on compound/in the bushes/garden nearby


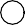

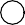


built bathroom/latrine building makeshift bathroom


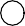

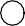

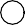


other bathing place

Other bathing place

How often do you bathe your feet? twice a day once a day not every day

Do you use soap when you bathe your feet? no sometimes always


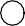

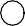

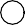


Where do you go (most of the time) to defecate? flush toilet ventilated pit latrine traditional latrine bush

How does your family dispose waste most of the time? open/roadside/in the garden/bush/farm

collected in pit collected and burned other - specify


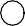

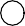

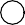

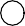

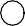

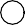

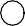

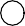


Other waste disposal

**Nutrition**

How many meals did the family eat yesterday?

Did you eat any of the following yesterday? (multiple
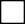
 starch (ugali, potato, cassava, cooking banana etc) choices possible)
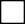
 vegetables (greens, tomatoes, etc)


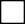
 meat, chicken or fish


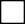
 fruits (papaya, banana, avocado, pineapple etc)
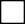
 dairy (milk etc)

**Caregiver behaviour (focus on the index child)**

What is the relationship of [name_of_index_child] to Child,


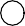

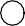

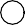

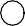

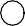


household head? Grandchild,

Niece/nephew, Adopted orphan Other-specify

Other relation

Does [name_of_index_child] have any disabilities ? Physical, mental, Both, None.

Does [name_of_index_child] have any current illnesses?
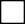
 none


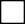
 Respiratory, chest, cough, nose
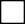
 diarrhea, stomach


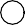

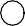

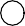

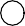


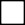
 skin rashes,
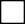
 eye problems
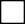
 ear problems
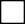
 headache


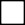
 Fever, malaria
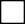
 Others

index child other illness

Does the index child have a chronic (meaning Yes

long-term) illness? No


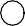

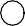


If the child has a chronic (long term) illnesses, what
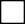
 HIV

kind?
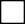
 Cancer


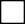
 Diabetes
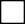
 Asthma


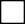
 Cystic fibrosis (affects lungs and digestive system)


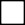
 Heart disease/defect


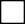
 Other organ (not heart) defects
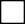
 Recurring diarrhoea/blood in stool
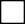
 DONT KNOW or not diagnosed (e.g. asthma, cancer, diabetes)

Did you/the mother have any birth complications with no

[name_of_index_child]? yes


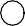

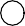


don't know

Is [name_of_index_child] sometimes absent from school Yes (for a day or longer) during term time? No

What are some of the reasons, why Jiggers

[name_of_index_child] is absent from school? Other Illness Lack of fees

Lack of uniform Home jobs

Truancy/naughty

Tired of school/ doesn't like it

(Do not read out the reason only select based on what the respondent says)

How often do you yourself (not somebody else) bathe twice a day, the younger children? once a day,

not every day never

At around what age of the children do you stop bathing/washing them yourself?

Do you or anyone else help or supervise no

[name_of_index_child] bathe? yes

don't know

If yes, who?

(e.g. self, sibling, house help, etc)

How often does [name_of_index_child] bathe? twice a day or more, once a day,

not every day never

don't know

Does [name_of_index_child] use soap for bathing? never sometimes always don't know

How much time do you spend talking with None

[name_of_index_child] each day? a little some a lot

Do you hug/cuddle [name_of_index_child]? Yes No

If yes, how often? Several times a day

once a day not every day

Do you think you did hug and cuddle Yes

[name_of_index_child] more often when he/she was No younger?

Did you spend more or less time with the child when more

he/she was younger? less

the same don't know

What did you do the last time [name_of_index_child] nothing

did something wrong? beat her/him,

talk to her/him calmly about mistake, send away/sit in one place,

shout at him/her other -specify

(Do not read out the reason only select based on what the respondent says)

Other

When was the last time you attended a parent meeting this term at school? last term

longer ago dont remember

When was the last time you talked to your child's this term

teacher about his/her school work? last term longer ago dont remember

Over the last week, did you check if Yes

[name_of_index_child] did her/his homework? No

Is there anyone in the household who read with Yes

[name_of_index_child] in the last 3 days? No

In the last school term did your child miss school to Yes

help with work in the house, in the farm or in the No family business?

Do you know the friends of [name_of_index_child] ? Yes No

Do you know the parents of your child's friends? Yes No

**Homestead and household characteristics**

How many adults (18 and above) live in your household including yourself?

How many children below the age or 5 live in your household?

How many children between 6 and 17 life in your household including the index child?

Is your household part of a larger homestead with Yes

other households? No

If yes, how many other family households are in the homestead?

How is the index child related to the other grandchild

households' heads? niece/nephew

other - specify

grandchild and niece/nephew

other relation

In total, how many adults (18 and above) live IN THE OTHER households?

In total, how many children (less than 18 years) live IN THE OTHER households?

Does your family/household keep any livestock (e.g Yes

cattle, goat, sheep, pigs (excluding birds)) No

Does any of the other household in the homestead Yes keep any livestock (e.g cattle, goat, sheep, pigs) No (excluding birds)

Are the livestock in the homestead kept together separately at night or separately (most of the time)? together

Where is the livestock kept? (also observe) at a central open place inside a shed

other specify

other location kept

Does your family/household keep any dogs or cats? Yes

No

Does any of the other household in the homestead Yes keep any dogs or cats? No

Do they roam freely between the households? Yes No

Do you (the index household) share any resources Receive support with the other households in your homestead (e.g. Give support

provide or receive financial support, sharing of Mutual

motorbike, provide or receive food etc? don't share any resources

What kind of support do you receive?

Does anyone in the other households have jiggers? Yes

No

How many of the other households are affected?

How many children are affected in all the other households together?

How many adults are affected in all the other households together?

**Request to be shown the INDEX household (support your questions with observations**

How many structures belong to the household? main house, kitchen house,

teenage boys' hut/small house/simba latrine/shower

other - specify

Other structures

How many sleeping areas are in the main house?

Specify the areas Adults only

Mixed adults & children bedroom

Children only bedroom (mixed boys/girls) Boys bedroom

Girls bedroom

Grandparent bedroom

Grandparent bedroom shared with (some) children Children in lounge

Other(specify)

(example: parent's bedroom children living room floor, etc)

How many adults sleep in the main house?

(if none enter 0)

How many children sleep in main house (but not in same bed as mother)

Observe the wall material of main house permanent stone or brick

semi-permanent mud/cement or mud/stone mudpalm leaves/grass/matt other-specify

other wall material

Observe the roof material of main house tiles

iron sheets

thatch (dry vegetation e.g grass, palm leaves etc) other-specify

Other roof material main house

Observe the state of repair of main house good

poor

very poor (holes in walls & roof)

State of sanitation inside the main house swept & tidy,

not swept, items scattered throughout other (specify

Other sanitation main house

Does anybody sleep in the kitchen? Yes No

Number of people sleeping in kitchen

How many adults (18 and above) sleep in kitchen?

(if none enter 0)

How many children sleep in the kitchen?

(if none enter 0)

Observe the wall material of the kitchen permanent stone or brick

semi-permanent mud/cement or mud/stone mud

palm leaves/grass/matt other-specify

Other wall kitchen

Observe the roof material of the kitchen tiles

iron sheets

thatch (dry vegetation e.g grass, palm leaves etc) other-specify

other roof kitchen

Observe the state of repair of the kitchen good

Poor

very poor (holes in walls & roof)

State of sanitation inside the kitchen swept & tidy,

not swept, items scattered throughout other (specify)

other sanitation state of kitchen

Does anybody sleep in the separate hut Yes

(simba/teenage boy's house etc.)? No

How many adults (18 and above) sleep in the hut?

How many children sleep in the hut?

Observe the wall material of the hut permanent stone or brick

semi-permanent mud/cement or mud/stone mud

palm leaves/grass/matt other-specify

other wall material hut

Observe the roof material of the hut tiles

iron sheets

thatch (dry vegetation e.g grass, palm leaves etc) other-specify

other roof material

Observe the state of repair of the hut good poor

very poor (holes in walls & roof)

State of sanitation inside the hut swept & tidy

not swept, items scattered throughout other (specify

other sanitation of hut

**Sleeping conditions of index child. Request to be shown where the index child sleeps**

In which structure does the index child sleep? Adults only

Mixed adults & children bedroom Children only bedroom (mixed boys/girls) Boys bedroom

Girls bedroom Grandparent bedroom

Grandparent bedroom shared with (some) children Children in lounge

Other(specify)

other sleeping place for index child

Does the index child share the sleeping place Yes

with other children? No

Observe (ask if unclear). Does the child sleep on Yes

a raised bed/structure? No

On what surface does the child sleep? mattress

rugs/old sheets/mosquito nets reed mats

nothing (bare frame or floor)

How tidy is the sleeping place of index child? swept & tidy,

not swept, items scattered throughout other (specify

other state of sleeping place

Is there any organic material in the same room Yes

(e.g. maize, beans, flour etc) No
