# Supplementary material for: Beyond poverty, tungiasis is associated with family characteristics and parenting behavior: a case control study in Kenya
Source: BMC Public Health. 2026 Jan 13;26:540. doi: 10.1186/s12889-026-26231-9 (PMC12888424; doi:10.1186/s12889-026-26231-9)
Supplement: Supplementary file 2 — Additional file 2. Pupil Questionnaire. Microsoft word file containing the questionnaire used to collect data February 2020-April 2021 during interviews of pupils aged 8 to 14 years in Kwale and Siaya counties of Kenya. Contains 5 pages of text, file size 263 Kb [file 12889_2026_26231_MOESM2_ESM.docx]

School Pupil Risk Factor Survey Tool

ID

Region BU


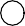

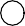

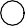


SI KW

School ID

Name of Index child

Index Child ID

Home village:

Date

Name of School

Class

Interviewer

Sex

Age

Tungiasis infection status Yes


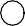

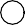


No

Observe: What shoes is the pupil wearing? none

**Individual risk factors**


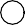

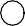

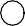

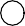


open slippers closed shoes

closed shoes and socks

Observe: Presence of school uniform none partially complete


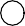

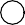

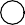


Observe: condition of uniform/other clothes torn moderate smart


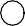

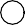

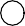


How do you come to school most days? walking motorbike taxi private motorbike tuktuk


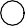

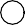

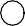

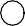

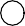

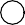


taxi private car

How many days did you go to school last week

If you missed school, why was that?

What adults do you live with most of the time? both parents single father single mother

**Parenting**


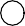

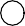

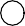

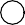

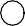

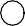

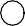

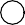

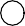


mother & stepfather father & stepmother grandparents

aunt & uncle no adults other/specify

Specify, adults you live with...

If you do NOT live with both of your parents, what live elsewhere for work is the reason? divorced/separated


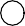

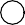

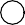

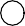

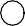


in hospital passed

don't know

Who cares for you?

Is there someone in your family who you fear?
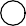
 Yes
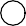
 No

Who do you fear?

Why?

Did your mother go to school? I don't know yes primary yes secondary no she didn't


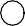

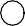

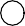

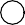


If you don't know, Can your mother read and write? no


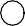

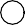

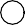


yes

don't know

Do you know if your father went to school? I don't know yes primary yes secondary no she didn't


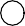

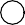

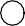

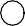


If you don't know, Can your father read and write? no yes


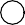

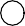


Does your father spend a lot of time away from the no home? yes


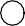

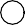


Does your mother spend a lot of time away from the no home? yes


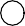

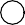


Do your parents know your friends? no yes


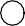

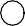


Do your parents know the parents of your friends? no yes


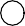

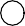


Do your parents attend school meetings and talk to never


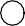

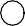

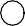


your teacher? sometimes

often

Do your parents make sure you do your homework? never


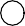

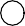

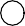


sometimes always

Do your parents help you with your homework? never sometimes always


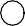

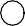

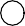


What do your parents do if you do something wrong? Beat you


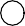

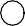

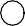

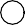

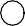

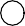


shout/yell/scream at you talk to you

send you away for some time make you sit in one place nothing

Who do you go to if you feel unwell or sad? mother father aunt uncle


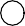

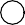

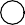

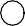

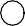

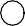

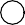

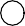


grandmother grandfather older sibling nobody

Is there anyone in your family who has been very no

**Socioeconomic risk factors**


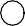

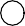


ill for some months? yes

If yes, who? father


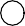

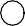

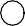

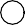

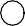

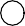


mother brother sister grandparent other

Is there anyone in your family with physical or no


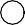

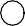


mental disability? yes

If yes, who? father


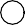

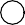

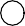

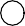

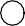

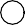


mother brother sister grandparent other

What do your parents do to provide you with food Have a job every day? casual labour


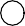

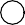

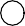


work on their own farm fishing

selling things in the market/village other (specify)

What is the material of the walls in your house? stone and cement

stone and mud mud and sticks makuti/grass/straw other

What is the material of the roof of your house? makuti/grass/straw mabati

other

How many sleeping rooms do you have?

Do you sleep on... bed with a mattress

traditional bed mattress on floor mat on floor

How many people sleep in the room with you?

How many meals did you eat yesterday?

Were you given the same amount of food as other no

children in your house? yes

Where do you get your water? tap on our compound

shared community tap/water kiosk nearby shared community tap/water kiosk far away collected by motorbike

community pump/ well river/stream/lake

rain puddles other specify

Specify where you get water...

How often do you wash your feet? twice a day once a day less often

Do you use soap when you wash your feet? never sometimes always

What kind of toilet do you use at home? flush toilet ventilated pit latrine traditional latrine bush

Does your family own a any of the following? radio TV

mobile phone bicycle

motorcycle car

solar lamp

none of these)

What animals does your family own? cow goat dog cat

**Animals**

chicken ducks sheep pig

none other

What animals spend time inside the house? cow goat dog cat

chicken ducks other none

sheep pig

other

What wild animals do you see in your None

compound/farm. monkeys

forest pigs rats

other specify

Specify the animal
