# Supplementary material for: Beyond poverty, tungiasis is associated with family characteristics and parenting behavior: a case control study in Kenya
Source: BMC Public Health. 2026 Jan 13;26:540. doi: 10.1186/s12889-026-26231-9 (PMC12888424; doi:10.1186/s12889-026-26231-9)
Supplement: Supplementary file 3 — Additional file 3. Microsoft word file containing the following sections on 26 pages of text, file size 175 Kb: S1 Polychoric principal component analysis for household socioeconomic status. Table S2. Household population characteristics by county. Table S3. Bivariable mixed effects logistic regression for household risk factors for tungiasis status for Kwale and Siaya. Table S4. Pupil characteristics by sex. Table S5. Bivariable mixed effects models for all pupils and boys and girls separately. Table S6. Factors associated with household socioeconomic status [file 12889_2026_26231_MOESM3_ESM.docx]

**Risk factors for tungiasis in Kwale and Siaya counties of Kenya: a case control study.**

Lynne Elson^1,2, *^, Abneel K. Matharu^3^, Naomi Riithi^3^, Paul Ouma^3^, Francis Mutebi^4^, Herman Feldmeier^5^, Jürgen Krücken^6,7^, Ulrike Fillinger^3^

**Additional File 3. Supporting Information**

Contents

[S1 Polychoric principal component analysis for household socioeconomic status 2](#_Toc199338098)

[Table S2 . Household population characteristics by county 7](#_Toc199338099)

[Table S3. Bivariable mixed effects logistic regression for household risk factors for tungiasis status for Kwale and Siaya. 12](#_Toc199338100)

[Table S4. Pupil characteristics by sex 19](#_Toc199338101)

[Table S5. Bivariable mixed effects models for all pupils and boys and girls separately 22](#_Toc199338102)

[Table S6. Factors associated with household socioeconomic status 26](#_Toc199338103)

## S1 Polychoric principal component analysis for household socioeconomic status

Stata code and outputs from conducting polychoric PCA for the households in both Kwale and Siaya.

polychoric radio0no1yes tv0no1yes mobile_phone0no1yes bicycle0no1yes motor_cycle0no1yes solar0no1yes own_livestock0no1yes

Polychoric correlation matrix

|  | radio | tv | mobile phone | bicycle | motorcycle | solar | livestock |
| --- | --- | --- | --- | --- | --- | --- | --- |
| radio | 1.000 |  |  |  |  |  |  |
| tv | 0.412 | 1.000 |  |  |  |  |  |
| mobile phone | 0.326 | 0.266 | 1.000 |  |  |  |  |
| bicycle | 0.270 | 0.141 | 0.231 | 1.000 |  |  |  |
| motorcycle | 0.292 | 0.350 | 0.350 | 0.188 | 1.000 |  |  |
| solar | 0.602 | 0.646 | 0.285 | 0.347 | 0.476 | 1.000 |  |
| livestock | 0.298 | 0.115 | 0.303 | 0.281 | 0.107 | 0.305 | 1.000 |

. display r(sum_w)

811

. global N=r(sum_w)

. matrix r=r(R)

. factormat r,n($N) factors (3)

(obs=811)

Factor analysis/correlation Number of obs = 811

Method: principal factors Retained factors = 3

Rotation: (unrotated) Number of params = 18

--------------------------------------------------------------------------

Factor | Eigenvalue Difference Proportion Cumulative

-------------+------------------------------------------------------------

Factor1 | 2.36836 2.00413 0.9948 0.9948

Factor2 | 0.36423 0.22759 0.1530 1.1478

Factor3 | 0.13665 0.14479 0.0574 1.2052

Factor4 | -0.00814 0.06803 -0.0034 1.2017

Factor5 | -0.07617 0.06362 -0.0320 1.1697

Factor6 | -0.13979 0.12455 -0.0587 1.1110

Factor7 | -0.26434 . -0.1110 1.0000

--------------------------------------------------------------------------

LR test: independent vs. saturated: chi2(21) = 1494.09 Prob>chi2 = 0.0000

Factor loadings (pattern matrix) and unique variances

-----------------------------------------------------------

Variable | Factor1 Factor2 Factor3 | Uniqueness

-------------+------------------------------+--------------

radio0no1yes | 0.6610 0.0480 -0.1162 | 0.5473

tv0no1yes | 0.6384 -0.3131 -0.0151 | 0.4942

mobile_pho~s | 0.4793 0.2075 0.2108 | 0.6828

bicycle0no~s | 0.4005 0.2567 -0.0525 | 0.7709

motor_cycl~s | 0.5287 -0.0901 0.2415 | 0.6541

solar0no1yes | 0.8393 -0.1471 -0.1128 | 0.2612

own_livest~s | 0.3871 0.3538 -0.0685 | 0.7203

-----------------------------------------------------------

. screeplot, yline(1)

. loadingplot

Figure 1. Plot of eigen values for each factor

Figure 2. Factor loadings for factor 1 against factor 2.

. predict factor3

(option regression assumed; regression scoring)

Scoring coefficients (method = regression)

--------------------------------------------

Variable | Factor1 Factor2 Factor3

-------------+------------------------------

radio0no1yes | 0.17552 0.10395 -0.12132

tv0no1yes | 0.15145 -0.32650 0.03652

mobile_phnes | 0.15919 0.19164 0.22500

bicycle0no1yes | 0.08223 0.22324 -0.03467

motor_cycles | 0.11527 -0.05329 0.31641

solar0no1yes | 0.48035 -0.19865 -0.24988

own_livestock | 0.08750 0.30593 -0.05276

--------------------------------------------

(variable means assumed 0; use means() option of factormat for nonzero means)

(variable std. deviations assumed 1; use sds() option of factormat to change)

. estat kmo

Kaiser-Meyer-Olkin measure of sampling adequacy

-----------------------

Variable | kmo

-------------+---------

radio0no1yes | 0.8146

tv0no1yes | 0.7151

mobile_phones | 0.7320

bicycle0no1yess | 0.8003

motor_cycles | 0.7693

solar0no1yes | 0.6756

own_livestock | 0.7263

-------------+---------

Overall | 0.7351

-----------------------

Figure 3. Frequency distribution of socio-economic factor

## Table S2 . Household population characteristics by county

**Categorical variables**

| **Variable** | **Category** | **All**  **(N^a^)** | **Kwale**  **N (%)** | **Siaya**  **N (%)** | **Chi^2^ P-value** |
| --- | --- | --- | --- | --- | --- |
| All |  | 769 | 418 | 351 |  |
| **Family structure** |  |  |  |  |  |
| Index pupil sex | Female | 363 | 214 (51.2) | 149 (42.7) | 0.019 |
|  | Male | 404 | 204 (48.8) | 200 (57.3) |  |
| HHH^b^ sex | Female | 176 | 85 (20.3) | 91 (25.9) | 0.066 |
|  | Male | 593 | 333 (79.7) | 260 (74.1) |  |
| Caregiver sex | Female | 713 | 389 (93.1) | 324 (92.3) | 0.688 |
|  | Male | 56 | 29 (6.9) | 27 (7.7) |  |
| Household religion | Muslim | 334 | 331 (79.4) | 3 (0.9) | <0.001 |
|  | Christian | 386 | 84 (20.1) | 302 (87.5) |  |
|  | Traditionist | 42 | 2 (0.5) | 40 (11.6) |  |
| Marital status of caregiver | Married | 593 | 326 (78.2) | 267 (77.4) | <0.001 |
|  | Widow | 113 | 43 (10.3) | 70 (20.3) |  |
|  | Single | 56 | 48 (11.5) | 8 (2.3) |  |
| Number adults | 0-1 | 115 | 37 (8.9) | 78 (22.2) | <0.001 |
|  | 2-4 | 386 | 207 (49.5) | 179 51.0) |  |
|  | >4 | 268 | 174 (41.6) | 94 (26.8) |  |
| Number under 5-year-olds | 0 | 286 | 140 (33.5) | 146 (41.7) | 0.004 |
|  | 1-2 | 430 | 256 (61.2) | 174 (49.7) |  |
|  | >2 | 52 | 22 (5.3) | 30 (8.6) |  |
| Number 6-17-year-olds | 1-3 | 463 | 232 (55.5) | 231 (65.8) | 0.004 |
|  | >3 | 306 | 186 (44.5) | 120 (34.2) |  |
| Index pupil relation to HHH | Child | 575 | 321 (76.8) | 254 (72.4) | 0.159 |
|  | Other | 194 | 97 (23.2) | 97 (27.6) |  |
| Index relation to caregiver | Child | 599 | 342 (81.8) | 257 (73.2) | <0.001 |
|  | Grandchild | 108 | 35 (8.4) | 73 (20.8) |  |
|  | other | 62 | 41 (9.8) | 21 (6.0) |  |
| Index pupil had other illness | No | 128 | 78 (18.7) | 50 (14.3) | 0.102 |
|  | Yes | 641 | 340 (81.3) | 301 (85.8) |  |
| Index pupil had birth complications | No | 626 | 358 (85.7) | 268 (77.0) | <0.001 |
|  | Yes | 101 | 58 (13.9) | 43 (12.4) |  |
|  | DK | 39 | 2 (0.5) | 37 (10.6) |  |
| Index pupil absent from school sometimes | No | 500 | 232 (55.5) | 268 (76.6) | <0.001 |
|  | Yes | 268 | 186 (45.5) | 82 (23.4) |  |
| Absent from school due to other illness | No | 615 | 302 (72.3) | 313 (89.2) | <0.001 |
|  | Yes | 154 | 116 (27.8) | 38 (10.8) |  |
| **Water, hygiene & sanitation** | | | | | |
| Water source | Not improved | 227 | 42 (10.1) | 185 (52.7) | <0.001 |
|  | Improved | 542 | 376 (89.9) | 166 (47.3) |  |
| Pay for water | No | 649 | 359 (85.9) | 290 (82.6) | 0.214 |
|  | Yes | 120 | 59 (14.1) | 61 (17.4) |  |
| Collect water from a long distance | No | 508 | 265 (63.4) | 243 (69.2) | 0.089 |
|  | Yes | 261 | 153 (36.6) | 108 (30.8) |  |
| Number of jerricans of water used/ day | 0-3 | 335 | 136 (32.5) | 199 (56.5) | 0.000 |
|  | 4-6 | 347 | 206 (49.3) | 141 (40.2) |  |
|  | >6 | 87 | 76 (18.2) | 11 (3.1) |  |
| Bathing place | Built stone | 66 | 23 (5.5) | 43 (12.3) | 0.001 |
|  | other | 703 | 395 (94.5) | 308 (87.7) |  |
| Caregiver frequency wash feet | Twice a day | 546 | 331 (79.2) | 215 (61.3) | <0.001 |
|  | Less | 223 | 87 (20.8) | 136 (38.8) |  |
| Caregiver soap use | Not always | 226 | 225 (53.8) | 1 (0.3) | <0.001 |
|  | Always | 543 | 193 (46.2) | 350 (99.7) |  |
| Age caregiver stops bathing children (years) | 11-15 | 123 | 83 (19.9) | 40 (11.4) | 0.005 |
|  | 6-10 | 433 | 228 (54.6) | 205 (58.4) |  |
|  | 1-5 | 213 | 107 (25.6) | 106 (30.2) |  |
| Index pupil frequency wash feet | Twice a day | 329 | 259 (62.0) | 70 (19.9) | <0.001 |
|  | Less | 440 | 159 (38.0) | 281 (80.1) |  |
| Index pupil soap use | Not always | 237 | 234 (56.0) | 3 (0.9) | <0.001 |
|  | Always | 532 | 184 (44.0) | 348 (99.2) |  |
| Toilet | Latrine | 573 | 252 (60.3) | 321 (91.5) | <0.001 |
|  | Open defecation | 196 | 166 (39.7) | 30 (8.6) |  |
| Waste disposal | open | 443 | 220 (52.6) | 223 (63.7) | <0.001 |
|  | pit | 264 | 149 (35.7) | 115 (32.9) |  |
|  | burned | 61 | 49 (11.7) | 12 (3.4) |  |
| **Household buildings** |  |  |  |  |  |
| Own land where house is | No | 709 | 379 (90.7) | 330 (94.0) | 0.085 |
|  | Yes | 60 | 39 (9.3) | 21 (6.0) |  |
| Own farmland | No | 356 | 260 (62.2) | 96 (27.4) | <0.001 |
|  | Yes | 412 | 158 (37.8) | 254 (72.6) |  |
| Part of a homestead | No | 488 | 254 (60.8) | 234 (66.9) | 0.081 |
|  | Yes | 280 | 164 (39.2) | 116 (33.1) |  |
| Other HH have jiggers | No | 241 | 133 (82.1) | 108 (93.1) | 0.008 |
|  | Yes | 37 | 29 (17.9) | 8 (6.9) |  |
| Walls of main house | Stone/ brick | 57 | 45 (10.8) | 12 (3.4) | <0.001 |
|  | Mud/mixed | 712 | 373 (89.2) | 339 (96.6) |  |
| Roof of main house | Natural | 296 | 264 (63.2) | 32 (9.1) | <0.001 |
|  | Other | 473 | 154 (36.8) | 319 (90.9) |  |
| Number of sleeping rooms in main house | 1 | 287 | 75 (17.9) | 212 (60.4) | <0.001 |
|  | 2 | 339 | 207 (49.5) | 132 (37.6) |  |
|  | >2 | 143 | 136 (32.5) | 7 (2.0) |  |
| Main house state of repair | Good | 634 | 295 (70.6) | 339 (96.9) | <0.001 |
|  | Not good | 135 | 123 (29.4) | 12 (3.4) |  |
| Main house sanitation | Clean & tidy | 511 | 182 (43.5) | 329 (94.0) | <0.001 |
|  | Not clean & tidy | 257 | 236 (56.5) | 21 (6.0) |  |
| Have separate kitchen building | No | 296 | 198 (47.4) | 98 (27.9) | <0.001 |
|  | Yes | 473 | 220 (52.6) | 253 (72.1) |  |
| People sleep in the kitchen | No | 328 | 200 (90.9) | 128 (50.8) | <0.001 |
|  | Yes | 144 | 20 (9.1) | 124 (49.2) |  |
| Kitchen hut state of repair | Good | 146 | 108 (49.1) | 38 (15.0) | <0.001 |
|  | Not good | 327 | 112 (50.9) | 215 (85.0) |  |
| Kitchen hut sanitation | Clean & tidy | 315 | 98 (44.6) | 217 (85.8) | <0.001 |
|  | Not clean & tidy | 158 | 122 (55.4) | 36 (14.2) |  |
| Have a separate hut for teenagers | No | 570 | 320 (76.6) | 250 (71.2) | 0.093 |
|  | Yes | 199 | 98 (23.4) | 101 (28.8) |  |
| People sleep in the separate hut | No | 12 | 4 (4.1) | 8 (8.1) | 0.241 |
|  | Yes | 185 | 94 (95.9) | 91 (91.9) |  |
| Separate hut state of repair | Good | 163 | 70 (71.4) | 93 (93.0) | <0.001 |
|  | Not good | 35 | 28 (28.6) | 7 (7) |  |
| Separate hut state of sanitation | Clean & tidy | 130 | 48 (49.0) | 82 (81.2) | <0.001 |
|  | Not clean & tidy | 69 | 50 (51) | 19 (18.8) |  |
| Where index child sleeps | In a room with adults | 204 | 159 (38.0) | 45 (12.8) | <0.001 |
|  | Mixed children’s room | 122 | 96 (22.9) | 26 (7.4) |  |
|  | Boys room | 129 | 77 (18.4) | 52 (14.8) |  |
|  | Girls room | 69 | 67 (16.0) | 2 (0.6) |  |
|  | with grandparents | 34 | 12 (2.9) | 22 (6.3) |  |
|  | Lounge | 105 | 7 (1.7) | 98 (27.9) |  |
|  | Kitchen | 99 | 0 | 99 (28.2) |  |
|  | Separate hut | 5 | 0 | 5 (1.4) |  |
|  | Neighbor’s hut | 2 | 0 | 2 (0.6) |  |
| Index child shares sleeping room with others | No | 125 | 53 (12.7) | 72 (20.7) | 0.003 |
|  | Yes | 641 | 365 (87.3) | 276 (79.3) |  |
| Index child sleeps on a raised bed | No | 299 | 75 (17.9) | 224 (64.4) | <0.001 |
|  | Yes | 467 | 343 (82.1) | 124 (35.6) |  |
| Surface index child sleeps on | mattress | 441 | 241 (57.7) | 200 (57.0) | 0.850 |
|  | rugs, mats etc | 328 | 177 (42.3) | 151 (43.0) |  |
| Index sleep place sanitation | swept & tidy | 456 | 157 (37.6) | 299 (85.7) | <0.001 |
|  | not swept & tidy | 311 | 261 (62.4) | 50 (14.3) |  |
| Index child floor of sleep room | Hard clay | 511 | 178 | 333 (94.9) | <0.001 |
|  | Loose soil | 102 | 84 | 18 (5.1) |  |
|  | Sand | 156 | 156 | 0 |  |
| Index child organic matter on floor | No | 580 | 281 (67.2) | 299 (85.7) | <0.001 |
|  | Yes | 187 | 137 (32.8) | 50 (14.3) |  |
| Household have a separate latrine/shower building | No | 548 | 306 (73.2) | 242 (69.0) | 0.194 |
|  | Yes | 221 | 112 (26.8) | 109 (31.1) |  |
|  |  |  |  |  |  |
| **Animals** |  |  |  |  |  |
| Own livestock | No | 387 | 229 (54.8) | 158 (45.0) | 0.007 |
|  | Yes | 382 | 189 (45.2) | 193 (55.0) |  |
| Other HH in homestead own livestock | No | 182 | 98 (59.8) | 84 (72.4) | 0.029 |
|  | Yes | 98 | 66 (40.2) | 32 (27.6) |  |
| Where livestock kept | open central place | 190 | 106 (46.7) | 84 (40.4) | 0.047 |
|  | closed shed | 221 | 114 (50.2) | 107 (51.4) |  |
|  | other | 24 | 7 (3.1) | 17 (8.2) |  |
| HH keeps cats/ dogs | No | 313 | 180 (43.1) | 133 (38.1) | 0.165 |
|  | Yes | 454 | 238 (56.9) | 216 (61.9) |  |
| Other HH in homestead keep cats/dogs | No | 197 | 104 (63.4) | 93 (80.9) | 0.002 |
|  | Yes | 82 | 60 (36.6) | 22 (19.1) |  |
| **Parenting** |  |  |  |  |  |
| Time spent with index pupil | None or a little | 378 | 181 (43.3) | 197 (56.1) | <0.001 |
|  | Some or a lot | 391 | 237 (56.7) | 154 (43.9) |  |
| Caregiver hugged index child | No | 540 | 312 (74.6) | 228 (65.0) | <0.001 |
|  | Yes | 229 | 106 (25.4) | 123 (35.0) |  |
| Hugged child more when younger | No | 91 | 9 (2.2) | 82 (23.4) | <0.001 |
|  | Yes | 677 | 409 (97.8) | 268 (76.6) |  |
| Discipline style | Other | 415 | 223 (53.4) | 192 (54.7) | 0.708 |
|  | Beat | 354 | 195 (46.7) | 159 (45.3) |  |
| When caregiver last attended a school meeting | Last term | 198 | 71 (17.0) | 127 (36.2) | <0.001 |
|  | Longer ago | 571 | 347 (83.0) | 224 (63.8) |  |
| When caregiver last talked to teacher | Last term | 185 | 63 (15.1) | 122 (34.8) | <0.001 |
|  | Longer ago | 584 | 355 (84.9) | 229 (65.2) |  |
| Caregiver checked child’s homework in last week | No | 398 | 266 (63.8) | 132 (37.9) |  |
|  | Yes | 367 | 151 (36.2) | 216 (62.1) |  |
| Someone read to the child in last 3 days | No | 315 | 186 (44.5) | 129 (36.8) | 0.030 |
|  | Yes | 454 | 232 (55.5) | 222 (63.3) |  |
| Caregiver knew index child’s friends | No | 143 | 81 (19.4) | 62 (17.7) | 0.555 |
|  | Yes | 625 | 337 (80.6) | 288 (82.3) |  |
| Caregiver knew the parents of child’s friends | No | 170 | 97 (23.3) | 73 (20.9) | 0.447 |
|  | Yes | 597 | 321 (76.8) | 276 (79.1) |  |
| Index child misses school to assist with house chores | No | 752 | 408 (97.6) | 344 (98.0) | 0.708 |
|  | Yes | 17 | 10 (2.4) | 7 (2.0) |  |

^a^ number of pupils, ^b^ head of household

**Numeric variables**

| **Variable** |  | **All** | **Kwale** | **Siaya** | **P-value^a^** |
| --- | --- | --- | --- | --- | --- |
| HHH^b^ age | Median (IQR) | 46 (39-57) | 45 (38-56) | 46 (39-58) | 0.777 |
| Caregiver age | Median (IQR) | 37 (31-45) | 36 (30-42) | 39 (32-48) | <0.001 |
| Index pupil age | Median (IQR) | 11 (9-12) | 11 (9-12) | 11 (9-12) | 0.992 |
| Number adults in HH^c^ | Median (IQR) | 2 (2-3) | 2 (2-3) | 2 (2-3) | <0.001 |
| Number <5 in HH | Median (IQR) | 1 (0-1) | 1 (0-1) | 1 (0-1) | 0.428 |
| Number 6-17 in HH | Median (IQR) | 3 (2-4) | 3 (2-4) | 3 (2-4) | <0.001 |
| Total family size | Median (IQR) | 6 (5-8) | 7 (5-8) | 6 (5-8) | <0.001 |
| Number adults sleep in main house | Median (IQR) | 2(2-2) | 2 (2-3) | 2 (1-2) | <0.001 |
| Number children sleep in main house | Median (IQR) | 2 (1-4) | 3 (2-4) | 1 (0-3) | <0.001 |
| Number adults sleep in kitchen | Median (IQR) | 0 (0-1) | 0 (0-1) | 0 (0-0.5) | <0.001 |
| Number children sleep in kitchen | Median (IQR) | 3 (2-4) | 2 (0.5-2) | 3 (2-4) | <0.001 |
| Number adults sleep in separate hut | Median (IQR) | 1 (0-1) | 1 (0-1) | 0 (0-1) | <0.001 |
| Number children sleep in separate hut | Median (IQR) | 2 (1-3) | 1 (0-3) | 2 (1-3) | 0.004 |
| Socio-economic status | Mean (sd) | 0.63 (0.13) | 0.56 (0.13) | 0.71 (0.35) | <0.001^d^ |

^a^ p value from Mann-Whitney test, ^b^ head of household, ^c^ household, ^d^ p-value from T-test

## Table S3. Bivariable mixed effects logistic regression for household risk factors for tungiasis status for Kwale and Siaya.

School as random effect Kwale n=418, 149 cases, Siaya n=351, 124 cases.

|  |  | **KWALE** | | | | | **SIAYA** | | | | |
| --- | --- | --- | --- | --- | --- | --- | --- | --- | --- | --- | --- |
| **Variables** | **Categories** | **N^a^** | **OR^b^** | **95% CI^c^** | | **P^d^** | **N^a^** | **OR^b^** | **95% CI^c^** | | **P^d^** |
| **Family structure** |  |  |  |  | |  |  |  |  |  |  |
| Index pupil sex | Female | 214 | 1 |  |  |  | 149 | 1 |  |  |  |
|  | Male | 204 | 1.67 | 1.12 | 2.51 | 0.012 | 200 | 1.05 | 0.66 | 1.67 | 0.828 |
| Index pupil age |  |  | 0.95 | 0.86 | 1.06 | 0.381 |  | 1 | 0.89 | 1.13 | 0.944 |
| HHH^e^ sex | Male | 333 | 1 |  |  |  | 260 | 1 |  |  |  |
|  | Female | 85 | 0.57 | 0.33 | 0.97 | 0.037 | 91 | 0.5 | 0.29 | 0.88 | 0.017 |
| HHH age |  |  | 1 | 0.99 | 1.02 | 0.587 |  | 1 | 0.98 | 1.02 | 0.841 |
| Caregiver sex | Male | 29 | 1 |  |  |  | 27 | 1 |  |  |  |
|  | Female | 389 | 0.57 | 0.27 | 1.21 | 0.145 | 324 | 0.79 | 0.34 | 1.84 | 0.586 |
| Caregiver age |  |  | 0.99 | 0.97 | 1.01 | 0.408 |  | 0.99 | 0.98 | 1.01 | 0.413 |
| Index pupil relation to HHH | Child | 321 | 1 |  |  |  | 254 | 1 |  |  |  |
|  | Other | 97 | 0.71 | 0.44 | 1.17 | 0.178 | 97 | 1.09 | 0.65 | 1.81 | 0.753 |
| Caregiver relation to index child | Parent | 342 | 1 |  |  |  | 257 | 1 |  |  |  |
|  | Grandparent | 35 | 0.93 | 0.45 | 1.93 | 0.844 | 73 | 0.73 | 0.41 | 1.33 | 0.307 |
|  | Other | 41 | 0.92 | 0.47 | 1.83 | 0.818 | 21 | 2.29 | 0.9 | 5.86 | 0.082 |
| Parent marital status | Married | 326 | 1 |  |  |  | 267 | 1 |  |  |  |
|  | Widowed | 43 | 0.37 | 0.17 | 0.83 | 0.015 | 70 | 0.57 | 0.31 | 1.06 | 0.078 |
|  | Other single | 48 | 0.89 | 0.47 | 1.68 | 0.727 | 8 | 2.35 | 0.75 | 7.37 | 0.144 |
| Household religion | Christian | 84 | 1 |  |  |  | 302 | 1 |  |  |  |
|  | Muslim | 331 | 0.88 | 0.53 | 1.46 | 0.618 | 3 | empty |  |  |  |
|  | Traditionist | 3 | 0.88 | 0.08 | 9.8 | 0.917 | 49 | 3.04 | 1.48 | 6.22 | 0.002 |
| Socioeconomic status |  |  | 1.19 | 0.68 | 2.08 | 0.542 |  | 0.82 | 0.42 | 1.59 | 0.554 |
| Number of adults in HH^f^ |  |  | 1.07 | 0.94 | 1.23 | 0.307 |  | 0.92 | 0.73 | 1.15 | 0.452 |
| Number children under 5 years in HH |  |  | 1.2 | 0.97 | 1.48 | 0.099 |  | 0.84 | 0.68 | 1.04 | 0.114 |
| Number children 6-17 years in HH |  |  | 1.08 | 0.96 | 1.21 | 0.186 |  | 1.18 | 0.98 | 1.42 | 0.074 |
| Index pupil had other illness | Yes | 78 | 1 |  |  |  | 128 | 1 |  |  |  |
|  | No illness | 340 | 1.41 | 0.83 | 2.4 | 0.209 | 641 | 1.02 | 0.52 | 2.01 | 0.949 |
| Index pupil birth complications | No | 358 | 1 |  |  |  | 268 | 1 |  |  |  |
|  | Yes | 58 | 0.65 | 0.35 | 1.21 | 0.173 | 43 | 0.95 | 0.45 | 1.99 | 0.884 |
|  | DK | 2 | 1.71 | 0.11 | 27.6 | 0.705 | 37 | 1.05 | 0.49 | 2.24 | 0.897 |
| Index pupil absent from school sometimes | No | 232 | 1 |  |  |  | 268 | 1 |  |  |  |
|  | Yes | 186 | 1.71 | 1.14 | 2.56 | 0.009 | 82 | 1.01 | 0.57 | 1.79 | 0.961 |
| Index pupil missed school due to other illness | No | 302 | 1 | 1 |  |  | 313 | 1 |  |  |  |
|  | Yes | 116 | 1.48 | 0.95 | 2.29 | 0.082 | 38 | 1.14 | 0.52 | 2.49 | 0.743 |
|  |  |  |  |  |  |  |  |  |  |  |  |
| **Water, hygiene & sanitation** | | | | | | |  |  |  |  |  |
| Water source | Not improved | 42 | 1 |  |  |  | 185 | 1 |  |  |  |
|  | Improved | 376 | 0.46 | 0.24 | 0.88 | 0.019 | 166 | 1.26 | 0.78 | 2.05 | 0.349 |
| Number jerrycans water used daily | 0-3 | 136 | 1 |  |  |  | 199 | 1 |  |  |  |
|  | 4-6 | 206 | 1.03 | 0.66 | 1.61 | 0.908 | 141 | 1.43 | 0.89 | 2.3 | 0.134 |
|  | >6 | 76 | 0.7 | 0.38 | 1.28 | 0.25 | 11 | 0.79 | 0.19 | 3.27 | 0.751 |
| Family pays for water used | No | 359 | 1 |  |  |  | 290 | 1 |  |  |  |
|  | Yes | 59 | 1.09 | 0.61 | 1.92 | 0.776 | 61 | 1.64 | 0.83 | 3.25 | 0.152 |
| Collect water from far away | No | 265 | 1 |  |  |  | 243 | 1 |  |  |  |
|  | Yes | 153 | 1.02 | 0.67 | 1.55 | 0.922 | 108 | 1.57 | 0.96 | 2.58 | 0.075 |
| Bathe place | Stone bathroom | 23 | 1 |  |  |  | 43 | 1 |  |  |  |
|  | Other | 395 | 1.28 | 0.52 | 3.19 | 0.592 | 308 | 1.06 | 0.53 | 2.15 | 0.866 |
| Caregiver frequency bathe feet | Twice a day | 331 | 1 |  |  |  | 215 | 1 |  |  |  |
|  | Less | 87 | 1.63 | 1.01 | 2.64 | 0.046 | 136 | 1.13 | 0.67 | 1.9 | 0.655 |
| Caregiver soap-use | Sometimes | 225 | 1 |  |  |  | 1 |  |  |  |  |
|  | Always | 193 | 0.72 | 0.48 | 1.08 | 0.111 | 351 | _ | _ | _ | _ |
| Age caregiver stops bathing children | 11-15 | 83 | 1 |  |  |  | 40 | 1 |  |  |  |
|  | 6-10 | 228 | 0.89 | 0.53 | 1.5 | 0.66 | 205 | 2.64 | 1.12 | 6.24 | 0.027 |
|  | 1-5 | 107 | 0.96 | 0.53 | 1.74 | 0.898 | 106 | 1.95 | 0.78 | 4.88 | 0.156 |
| Index pupil frequency bathe feet | Twice a day | 259 | 1 |  |  |  | 70 | 1 |  |  |  |
|  | Less | 159 | 1.72 | 1.14 | 2.58 | 0.010 | 281 | 1.01 | 0.57 | 1.77 | 0.986 |
| Index pupil soap use | Always | 184 | 1 |  |  |  | 348 | 1 |  |  |  |
|  | Not always | 234 | 1.27 | 0.85 | 1.9 | 0.251 | 3 | 1.45 | 0.12 | 18.27 | 0.771 |
| Toilet | Pit latrine | 252 | 1 |  |  |  | 321 | 1 |  |  |  |
|  | Open defecation | 166 | 1.13 | 0.75 | 1.7 | 0.555 | 30 | 0.38 | 0.15 | 1.01 | 0.053 |
| Waste disposal method | Open | 220 | 1 |  |  |  | 223 | 1 |  |  |  |
|  | Pit | 149 | 0.79 | 0.51 | 1.23 | 0.299 | 115 | 0.92 | 0.55 | 1.54 | 0.754 |
|  | Burned | 49 | 0.78 | 0.41 | 1.51 | 0.47 | 12 | 0.49 | 0.12 | 2.02 | 0.323 |
|  |  |  |  |  |  |  |  |  |  |  |  |
| **Parenting** |  |  |  |  |  |  |  |  |  |  |  |
| Caregiver hugs index child | No | 312 | 1 |  |  |  | 228 | 1 |  |  |  |
|  | Yes | 106 | 1.01 | 0.64 | 1.6 | 0.96 | 123 | 0.78 | 0.48 | 1.28 | 0.333 |
| How much time caregiver spent with index pupil | None or little | 181 | 1 |  |  |  | 197 | 1 |  |  |  |
|  | Some or a lot | 237 | 1.07 | 0.71 | 1.6 | 0.754 | 154 | 0.8 | 0.49 | 1.32 | 0.381 |
| Discipline style | Other method | 223 | 1 |  |  |  | 192 | 1 |  |  |  |
|  | Beat child | 195 | 0.9 | 0.6 | 1.35 | 0.608 | 159 | 0.97 | 0.61 | 1.53 | 0.883 |
| When caregiver last attended a parent meeting at school | Last term | 71 | 1 |  |  |  | 127 | 1 |  |  |  |
|  | Longer ago | 347 | 0.82 | 0.49 | 1.39 | 0.465 | 224 | 0.74 | 0.42 | 1.31 | 0.298 |
| When caregiver last talked to teacher | Last term | 63 | 1 |  |  |  | 122 | 1 |  |  |  |
|  | Longer ago | 355 | 0.88 | 0.51 | 1.54 | 0.66 | 229 | 0.81 | 0.47 | 1.41 | 0.453 |
| Caregiver checked index pupil homework in last week | No | 266 | 1 |  |  |  | 132 | 1 |  |  |  |
|  | Yes | 151 | 0.97 | 0.64 | 1.48 | 0.9 | 216 | 0.92 | 0.56 | 1.5 | 0.736 |
| Someone reads with index pupil | No | 186 | 1 |  |  |  | 129 | 1 |  |  |  |
|  | Yes | 232 | 0.69 | 0.46 | 1.04 | 0.074 | 222 | 0.92 | 0.56 | 1.5 | 0.735 |
| Caregiver knows index pupil's friends | No | 81 | 1 |  |  |  | 62 | 1 |  |  |  |
|  | Yes | 337 | 0.76 | 0.46 | 1.25 | 0.287 | 288 | 0.54 | 0.3 | 0.97 | 0.041 |
| Caregiver knows index pupil's friends' parents | No | 97 | 1 |  |  |  | 73 | 1 |  |  |  |
|  | Yes | 321 | 0.78 | 0.49 | 1.24 | 0.285 | 276 | 0.64 | 0.37 | 1.11 | 0.114 |
| **Household buildings** |  |  |  |  |  |  |  |  |  |  |  |
| Own the land where house is | No | 379 | 1 |  |  |  | 330 | 1 |  |  |  |
|  | Yes | 39 | 1.29 | 0.66 | 2.52 | 0.462 | 21 | 2.11 | 0.82 | 5.42 | 0.121 |
| Own farming land | No | 260 | 1 |  |  |  | 96 | 1 |  |  |  |
|  | Yes | 158 | 1.03 | 0.68 | 1.56 | 0.886 | 254 | 1.48 | 0.87 | 2.54 | 0.15 |
| HH is part of larger homestead | No | 254 | 1 |  |  |  | 234 | 1 |  |  |  |
|  | Yes | 164 | 0.86 | 0.57 | 1.3 | 0.47 | 116 | 1.29 | 0.79 | 2.09 | 0.311 |
| Numb of HH in homestead |  |  | 1.36 | 0.99 | 1.87 | 0.061 |  | 1.13 | 0.91 | 1.4 | 0.279 |
| HH has a separate kitchen building | No | 198 | 1 |  |  |  | 98 | 1 |  |  |  |
|  | Yes | 220 | 0.87 | 0.58 | 1.29 | 0.484 | 253 | 1.19 | 0.7 | 2.03 | 0.517 |
| HH has a separate hut for teenagers | No | 320 | 1 |  |  |  | 250 | 1 |  |  |  |
|  | Yes | 98 | 1.58 | 0.99 | 2.51 | 0.053 | 101 | 0.98 | 0.59 | 1.64 | 0.949 |
| HH has a separate latrine/shower building | No | 306 | 1 |  |  |  | 242 | 1 |  |  |  |
|  | Yes | 112 | 0.81 | 0.51 | 1.28 | 0.366 | 109 | 1.32 | 0.78 | 2.24 | 0.302 |
| Number of sleep rooms in main house | 1 | 75 | 1 |  |  |  | 7 | 1 |  |  |  |
|  | 2 | 207 | 0.67 | 0.39 | 1.15 | 0.15 | 212 | 0.72 | 0.15 | 3.5 | 0.679 |
|  | >2 | 136 | 0.73 | 0.41 | 1.31 | 0.291 | 132 | 1.26 | 0.26 | 6.24 | 0.773 |
| Number of adults sleep in main house |  |  | 0.99 | 0.79 | 1.25 | 0.933 |  | 1.13 | 0.73 | 1.75 | 0.584 |
| Number of children sleep in main house |  |  | 1.12 | 1 | 1.26 | 0.059 |  | 1.07 | 0.93 | 1.24 | 0.333 |
| Main house wall material | Stone/brick | 45 | 1 |  |  |  | 12 | 1 |  |  |  |
|  | Mud/mixed | 373 | 1.12 | 0.58 | 2.16 | 0.732 | 339 | 0.86 | 0.25 | 2.93 | 0.804 |
| Main house roof material | Natural | 264 | 1 |  |  |  | 32 | 1 |  |  |  |
|  | Other | 154 | 0.8 | 0.53 | 1.22 | 0.3 | 319 | 0.99 | 0.45 | 2.24 | 0.997 |
| Main house state of repair | Good | 295 | 1 |  |  |  | 339 | 1 |  |  |  |
|  | Not good | 123 | 1.01 | 0.65 | 1.56 | 0.972 | 12 | 0.44 | 0.08 | 2.29 | 0.327 |
| Main house sanitation | Swept & tidy | 182 | 1 |  |  |  | 329 | 1 |  |  |  |
|  | Not swept & tidy | 236 | 1.4 | 0.93 | 2.11 | 0.105 | 21 | 0.7 | 0.25 | 1.96 | 0.501 |
| People sleep in the kitchen building | No | 200 | 1 |  |  |  | 128 | 1 |  |  |  |
|  | Yes | 20 | 0.82 | 0.3 | 2.21 | 0.686 | 124 | 0.64 | 0.37 | 1.12 | 0.116 |
| Number of adults sleep in kitchen building |  |  | 0.8 | 0.26 | 2.44 | 0.694 |  | 0.88 | 0.44 | 1.76 | 0.712 |
| Number of children sleep in kitchen building |  |  | 1.62 | 0.74 | 3.53 | 0.224 |  | 0.77 | 0.55 | 1.07 | 0.117 |
| Index pupil sleep place | With adults | 159 | 1 |  |  |  | 45 | 1 |  |  |  |
|  | Mixed kids | 96 | 1.58 | 0.93 | 2.67 | 0.088 | 26 | 1.23 | 0.44 | 3.49 | 0.691 |
|  | Boys only room | 77 | 1.67 | 0.96 | 2.93 | 0.071 | 52 | 0.88 | 0.37 | 2.13 | 0.785 |
|  | Girls only room | 67 | 0.78 | 0.41 | 1.47 | 0.438 | 2 | 1.19 | 0.06 | 23.79 | 0.91 |
|  | Grandparents room | 12 | 0.42 | 0.09 | 2 | 0.279 | 22 | 1.14 | 0.38 | 3.39 | 0.812 |
|  | Lounge | 7 | 1.59 | 0.34 | 7.36 | 0.554 | 98 | 1.06 | 0.48 | 2.3 | 0.892 |
|  | Kitchen | 0 |  |  |  |  | 99 | 0.84 | 0.38 | 1.84 | 0.666 |
|  | Separate hut | 0 |  |  |  |  | 5 | 1.49 | 0.2 | 10.93 | 0.696 |
|  | Neighbor’s hut | 0 |  |  |  |  | 2 | _ |  |  |  |
| Index pupil shares sleep room with others | No | 53 | 1 |  |  |  | 72 | 1 |  |  |  |
|  | Yes | 365 | 0.99 | 0.54 | 1.81 | 0.974 | 276 | 0.9 | 0.51 | 1.6 | 0.724 |
| Index pupil sleeps on a raised bed | No | 75 | 1 |  |  |  | 224 | 1 |  |  |  |
|  | Yes | 343 | 0.74 | 0.45 | 1.24 | 0.257 | 124 | 0.88 | 0.54 | 1.43 | 0.604 |
| Surface index pupil sleeps on | Mattress | 241 | 1 |  |  |  | 200 | 1 |  |  |  |
|  | Rugs, mats etc | 177 | 1.73 | 1.15 | 2.6 | 0.008 | 151 | 0.95 | 0.59 | 1.53 | 0.843 |
| Sanitation of index pupil's room | Swept & tidy | 157 | 1 |  |  |  | 299 | 1 |  |  |  |
|  | Not swept & tidy | 261 | 1.57 | 1.03 | 2.4 | 0.036 | 50 | 1.04 | 0.54 | 2.01 | 0.911 |
| Organic material on floor of index pupil's room | No | 281 | 1 |  |  |  | 299 | 1 |  |  |  |
|  | Yes | 137 | 1.01 | 0.66 | 1.54 | 0.971 | 50 | 1.56 | 0.82 | 3 | 0.178 |
| Floor material of index pupil's sleep room | Hard clay | 178 | 1 |  |  |  | 333 | 1 |  |  |  |
|  | Loose soil | 84 | 1.6 | 0.93 | 2.76 | 0.087 | 18 | 1.01 | 0.35 | 2.92 | 0.989 |
|  | Sand | 156 | 1.56 | 0.99 | 2.45 | 0.056 | 0 | _ |  |  |  |
|  |  |  |  |  |  |  |  |  |  |  |  |
| **Animals** |  |  |  |  |  |  |  |  |  |  |  |
| HH owns livestock | No | 229 | 1 |  |  |  | 158 | 1 |  |  |  |
|  | Yes | 189 | 0.83 | 0.56 | 1.24 | 0.37 | 193 | 1.05 | 0.66 | 1.67 | 0.846 |
| Other HHs in homestead own livestock | No | 98 | 1 |  |  |  | 84 | 1 |  |  |  |
|  | Yes | 66 | 0.7 | 0.36 | 1.36 | 0.291 | 32 | 1.95 | 0.82 | 4.65 | 0.133 |
|  |  |  |  |  |  |  | 94 | 1 |  |  |  |
|  |  |  |  |  |  |  | 107 | 1.02 | 0.52 | 1.97 | 0.958 |
|  |  |  |  |  |  |  | 17 | 0.79 | 0.23 | 2.68 | 0.706 |
| HH keeps cats or dogs | No | 180 | 1 |  |  |  | 133 | 1 |  |  |  |
|  | Yes | 238 | 0.72 | 0.48 | 1.07 | 0.107 | 216 | 1.31 | 0.81 | 2.11 | 0.275 |

^a^ number of households, ^b^ odds ratio, ^c^ confidence interval, ^d^ p value, ^e^ household head, ^f^household

## Table S4. Pupil characteristics by sex

| **Variable** | **Category** | **All**  **N^a^ (%)** | **Female**  **N (%)** | **Male**  **N (%)** | **p-value^b^** |
| --- | --- | --- | --- | --- | --- |
| All |  | 395 | 168 | 227 |  |
| Tungiasis status | Uninfected | 197 | 111 (66.1) | 86 (37.9) | 0.000 |
|  | Infected | 198 | 57 (33.9) | 141 (62.1) |  |
| Region | Kwale | 193 | 88 (52.4) | 105 (46.3) | 0.229 |
|  | Siaya | 202 | 80 (47.6) | 122 (53.7) |  |
| Shoes worn on the day | Closed | 95 | 43 (25.6) | 52 (22.9) | 0.537 |
|  | Other | 300 | 125 (74.4) | 175 (77.1) |  |
| Uniform worn on the day | Complete | 70 | 32 (19.1) | 38 (16.7) | 0.553 |
|  | Other | 325 | 136 (81.0) | 189 (83.3) |  |
| Uniform condition | Torn & dirty | 66 | 18 (17.3) | 48 (37.8) | 0.001 |
|  | Other | 165 | 86 (82.7) | 79 (62.2) |  |
| Adults live with | Both parents | 252 | 113 (67.3) | 139 (61.2) | 0.218 |
|  | Other | 143 | 55 (32.7) | 88 (38.8) |  |
| HHH^c^ sex | Female | 87 | 35 (21.7) | 52 (23.0) | 0.768 |
|  | Male | 300 | 126 (78.3) | 174 (58.0) |  |
| Caregiver sex | Female | 354 | 151 (93.8) | 203 (89.8) | 0.169 |
|  | Male | 33 | 10 (6.2) | 23 (10.2) |  |
| Caregiver relationship to child | Mother | 295 | 128 (76.2) | 167 (73.9) | 0.603 |
|  | Other | 99 | 40 (23.8) | 59 (26.1) |  |
| Who child went to when unwell | Parent | 191 | 86 (51.5 | 105 (47.1) | 0.388 |
|  | Other | 199 | 81 (48.5) | 118 (52.9) |  |
| Mother’s education | None | 38 | 20 (11.9) | 18 (7.9) | 0.163 |
|  | Don’t know | 159 | 58 (34.5) | 101 (44.5) |  |
|  | Primary | 145 | 68 (40.5) | 77 (33.9) |  |
|  | Secondary | 53 | 22 (13.1) | 31 (13.7) |  |
| Father’s education | None | 13 | 6 (3.6) | 7 (3.1) | 0.954 |
|  | Don’t know | 176 | 74 (44.3) | 102 (45.1) |  |
|  | Primary | 125 | 55 (32.9) | 70 (31.0) |  |
|  | Secondary | 79 | 32 (19.2) | 47 (20.8) |  |
| Father away a lot | No | 121 | 52 (31.1) | 69 (31.8) | 0.890 |
|  | Yes | 263 | 115 (68.9) | 148 (68.2) |  |
| Mother away a lot | No | 274 | 119 (71.7) | 155 (68.9) | 0.550 |
|  | Yes | 117 | 47 (28.3) | 70 (31.1) |  |
| Family member ill for some months | No | 241 | 107 (63.7) | 134 (59.0) | 0.348 |
|  | Yes | 154 | 61 (36.3) | 93 (41.0) |  |
| Family member had a disability | No | 365 | 154 (92.8) | 211 (93.0) | 0.945 |
|  | Yes | 28 | 12 (7.2) | 16 (7.0) |  |
| Child feared a family member | No | 264 | 104 (61.9) | 160 (70.8) | 0.063 |
|  | Yes | 130 | 64 (38.1) | 66 (29.2) |  |
| Family member feared | Father | 41 | 26 (40.6) | 15 (22.7) | 0.060 |
|  | Mother | 22 | 11 (17.2) | 11 (6.7) |  |
|  | Both parents | 14 | 8 (12.5) | 6 (9.1) |  |
|  | Brother | 14 | 3 (4.7) | 11 (16.7) |  |
|  | Others | 39 | 16 (25.0) | 23 (34.9) |  |
| Parents knew child’s friends | No | 42 | 14 (8.3) | 28 (12.3) | 0.202 |
|  | Yes | 353 | 154 (91.7) | 199 (87.7) |  |
| Parents knew child’s friends’ parents | No | 62 | 24 (14.3) | 38 (16.7) | 0.507 |
|  | Yes | 333 | 144 (85.7) | 189 (83.3) |  |
| Parents attend school meetings | No | 189 | 82 (49.1) | 107 (47.1) | 0.700 |
|  | Yes | 205 | 85 (50.9) | 120 (52.9) |  |
| Parents’ discipline style | Beat | 266 | 113 (67.3) | 153 (67.4) | 0.977 |
|  | Other | 129 | 55 (32.7) | 74 (32.6) |  |
| Bed child slept on | Raised bed | 208 | 95 (56.6) | 113 (49.8) | 0.183 |
|  | On floor | 187 | 73 (43.5) | 114 (50.2) |  |
| Frequency child washed feet | Twice a day | 293 | 135 (81.8) | 158 (69.9) | 0.007 |
|  | Less often | 98 | 30 (18.2) | 68 (30.1) |  |
| Soap-use for feet washing | Always | 150 | 67 (39.9) | 83 (36.7) | 0.524 |
|  | Less | 244 | 101 (60.1) | 143 (63.3) |  |
| Toilet used | Open defecation | 72 | 33 (19.6) | 39 (17.2) | 0.531 |
|  | Latrine | 323 | 135 (80.4) | 188 (82.8) |  |
| Wall materials | Mud | 302 | 124 (73.8) | 178 (78.4) | 0.286 |
|  | Other | 93 | 44 (26.2) | 49 (21.6) |  |
| Roof materials | Natural | 139 | 67 (40.0) | 72 (32.3) | 0.110 |
|  | Iron sheet | 251 | 100 (60.0) | 151 (67.7) |  |
| Child received equal food to others | No | 23 | 6 (3.6) | 17 (7.5) | 0.100 |
|  | Yes | 372 | 162 (96.4) | 210 (92.5) |  |
| Orphaned | No | 369 | 159 (94.6) | 210 (92.5) | 0.398 |
|  | Yes | 26 | 9 (5.4) | 17 (7.5) |  |
| Caregiver depression | No | 253 | 110 (65.5) | 143 (63.0) | 0.611 |
|  | Yes | 142 (35.9) | 58 (34.5) | 84 (37.0) |  |
| Complications during child’s delivery | No | 309 | 128 (84.2) | 181 (87.4) | 0.383 |
|  | Yes | 50 | 24 (15.8) | 26 (12.6) |  |
| Time caregiver spent with child | Not a lot | 273 | 106 (65.8) | 167 (73.9) | 0.087 |
|  | A lot | 114 | 55 (34.2) | 59 (26.1) |  |
| Caregiver hugged child | No | 277 | 110 (68.3) | 167 (73.9) | 0.231 |
|  | Yes | 110 | 51 (31.7) | 59 (26.1) |  |
| Number people shared sleeping room with child | >1 | 246 | 106 (63.1) | 140 (61.7) | 0.773 |
|  | 0-1 | 149 | 62 (36.9) | 87 (38.3) |  |
| **Numeric variables** |  |  |  |  |  |
| Pupil age | Mean (sd) |  | 10.8 (2.0) | 10.7 (1.8) | 0.618^d^ |
| HHH age | Mean (sd) |  | 47.8 (13.3) | 49.3 (12.5) | 0.255^d^ |
| Caregiver age | Mean (sd) |  | 39.0 (12.2) | 40.1 (12.1) | 0.357^d^ |
| SES | Mean (sd) |  | 0.79 (0.37) | 0.83 (0.33) | 0.285^d^ |
| Parental stress score | Median (IQR) |  | 46 (41-50) | 46 (40-50) | 0.530^e^ |

^a^ Number of pupils, ^b^ p value from Chi^2^ test, ^c^ head of household, ^d^ p-value from T-test, ^e^ p-value from Mann-Whitney test

## Table S5. Bivariable mixed effects models for all pupils and boys and girls separately

**Univariable mixed effect logistic regression**, school as random effect

bold black font=significant for both girls & boys, red significant for girls only, blue significant for boys only

|  |  | **ALL PUPILS** | | | | | **GIRLS** | | | | | | | **BOYS** | | | | | | | | |
| --- | --- | --- | --- | --- | --- | --- | --- | --- | --- | --- | --- | --- | --- | --- | --- | --- | --- | --- | --- | --- | --- | --- |
| **Variables** | **Categories** | **N^a^** | **OR^b^** | **95% CI^c^** | | **P^d^** | **N^a^** | **OR^b^** | **95% CI^c^** | | | **P^d^** | | **N** | | **OR** | | **95% CI** | | | **P** | |
| Pupil sex | Female | 168 | 1 |  |  |  |  |  |  |  |  | |  | |  | |  | |  |  | |  |
|  | Male | 227 | 3.19 | 2.1 | 4.84 | <0.001 |  |  |  |  |  | |  | |  | |  | |  |  | |  |
| Region | Kwale | 193 | 1 |  |  |  | 88 | 1 |  |  |  | | 105 | | 1 | |  | |  |  | |  |
|  | Siaya | 202 | 1.05 | 0.71 | 1.56 | 0.801 | 80 | 1.22 | 0.64 | 2.31 | 0.545 | | 122 | | 0.81 | | 0.47 | | 1.39 | 0.446 | |  |
| Pupil age |  |  | 0.84 | 0.76 | 0.94 | 0.002 |  | 0.81 | 0.69 | 0.96 | 0.014 | |  | | 0.86 | | 0.74 | | 1.00 | 0.047 | |  |
| Shoes worn | Closed | 95 | 1 |  |  |  | 43 | 1 |  |  |  | | 52 | | 1 | |  | |  |  | |  |
|  | Other | 300 | 1.62 | 1.01 | 2.58 | 0.043 | 125 | 2.35 | 1.04 | 5.34 | 0.040 | | 175 | | 1.27 | | 0.68 | | 2.39 | 0.455 | |  |
| Uniform worn | Complete | 70 | 1 |  |  |  | 32 | 1 |  |  |  | | 38 | | 1 | |  | |  |  | |  |
|  | Not complete | 325 | 1.65 | 0.97 | 2.78 | 0.063 | 136 | 6.37 | 1.85 | 21.94 | 0.003 | | 189 | | 0.83 | | 0.40 | | 1.72 | 0.609 | |  |
| Adults child lives with | Both parents | 252 | 1 |  |  |  | 113 | 1 |  |  |  | | 139 | | 1 | |  | |  |  | |  |
|  | Others | 143 | 1.26 | 0.84 | 1.91 | 0.266 | 55 | 0.92 | 0.47 | 1.83 | 0.819 | | 88 | | 1.41 | | 0.81 | | 2.47 | 0.224 | |  |
| Primary caregiver | Mother | 295 | 1 |  |  |  | 128 | 1 |  |  |  | | 167 | | 1 | |  | |  |  | |  |
|  | Other | 99 | 1.14 | 0.73 | 1.81 | 0.562 | 40 | 1.41 | 0.68 | 2.95 | 0.354 | | 59 | | 0.95 | | 0.52 | | 1.74 | 0.864 | |  |
| Who child goes to when unwell | A parent | 191 | 1 |  |  |  | 86 | 1 |  |  |  | | 105 | | 1 | |  | |  |  | |  |
|  | Others | 199 | 0.96 | .65 | 1.43 | 0.841 | 81 | 0.98 | 0.52 | 1.87 | 0.958 | | 118 | | 0.86 | | 0.50 | | 1.47 | 0.577 | |  |
| HHH^e^ sex | female | 87 | 1 |  |  |  | 35 | 1 |  |  |  | | 52 | | 1 | |  | |  |  | |  |
|  | male | 300 | 1.05 | 0.65 | 1.69 | 0.839 | 126 | 2.01 | 0.84 | 4.78 | 0.115 | | 174 | | 0.76 | | 0.39 | | 1.46 | 0.405 | |  |
| Caregiver sex | female | 354 | 1 |  |  |  | 151 | 1 |  |  |  | | 203 | | 1 | |  | |  |  | |  |
|  | male | 33 | 2.55 | 1.19 | 5.50 | 0.017 | 10 | 3.12 | 0.84 | 11.57 | 0.089 | | 23 | | 2.34 | | 0.84 | | 6.56 | 0.105 | |  |
| Age of HHH |  |  |  |  |  |  |  | 1.00 | 0.98 | 1.03 | 0.775 | |  | | 1.01 | | 0.99 | | 1.04 | 0.210 | |  |
| Age of caregiver |  |  | 1.00 | 0.98 | 1.02 | 0.94 |  | 1.00 | 0.98 | 1.03 | 0.767 | |  | | 0.99 | | 0.97 | | 1.02 | 0.527 | |  |
| Child feared a family member | No | 264 | 1 |  |  |  | 104 | 1 |  |  |  | | 160 | | 1 | |  | |  |  | |  |
|  | Yes | 130 | 1.03 | 0.68 | 1.57 | 0.886 | 64 | 0.73 | 0.38 | 1.43 | 0.363 | | 66 | | 1.74 | | 0.94 | | 3.22 | 0.080 | |  |
| Father away a lot | No | 121 | 1 |  |  |  | 52 | 1 |  |  |  | | 69 | | 1 | |  | |  |  | |  |
|  | Yes | 263 | 0.98 | 0.63 | 1.5 | 0.913 | 115 | 1.25 | 0.62 | 2.51 | 0.538 | | 148 | | 0.83 | | 0.46 | | 1.50 | 0.533 | |  |
| Mother away a lot | No | 274 | 1 |  |  |  | 119 | 1 |  |  |  | | 155 | | 1 | |  | |  |  | |  |
|  | Yes | 117 | 1.36 | 0.88 | 2.11 | 0.161 | 47 | 1.94 | 0.97 | 3.89 | 0.063 | | 70 | | 1.04 | | 0.58 | | 1.86 | 0.895 | |  |
| Parents know child’s friends | No | 42 |  |  |  |  | 14 | 1 |  |  |  | | 28 | | 1 | |  | |  |  | |  |
|  | Yes | 353 | 1.12 | 0.59 | 2.12 | 0.731 | 154 | 0.66 | 0.22 | 2.00 | 0.464 | | 199 | | 1.76 | | 0.80 | | 3.91 | 0.162 | |  |
| Parents know friends’ parents | No | 62 |  |  |  |  | 24 | 1 |  |  |  | | 38 | | 1 | |  | |  |  | |  |
|  | Yes | 333 | 1.17 | 0.68 | 2.02 | 0.566 | 144 | 0.68 | 0.28 | 1.64 | 0.389 | | 189 | | 1.82 | | 0.90 | | 3.68 | 0.094 | |  |
| Parents attend school meetings | Often | 189 | 1 |  |  |  | 82 | 1 |  |  |  | | 107 | | 1 | |  | |  |  | |  |
|  | Not often | 205 | 1.6 | 1.07 | 2.38 | 0.021 | 85 | 2.28 | 1.17 | 4.42 | 0.015 | | 120 | | 1.30 | | 0.76 | | 2.22 | 0.343 | |  |
| Discipline style | Other | 129 | 1 |  |  |  | 55 | 1 |  |  |  | | 74 | | 1 | |  | |  |  | |  |
|  | Beat | 266 | 0.90 | 0.59 | 1.37 | 0.618 | 113 | 1.80 | 0.88 | 3.68 | 0.108 | | 153 | | 0.77 | | 0.43 | | 1.37 | 0.376 | |  |
| Family member ill some months | No | 241 | 1 |  |  |  | 107 | 1 |  |  |  | | 134 | | 1 | |  | |  |  | |  |
|  | Yes | 154 | 1.08 | 0.72 | 1.62 | 0.710 | 61 | 0.92 | 0.47 | 1.80 | 0.813 | | 93 | | 1.10 | | 0.64 | | 1.90 | 0.731 | |  |
| Bed child slept on | Raised bed | 208 | 1 |  |  |  | 71 | 1 |  |  |  | | 78 | | 1 | |  | |  |  | |  |
|  | On floor | 187 | 1.66 | 1.09 | 2.51 | 0.018 | 88 | 1.03 | 0.54 | 1.95 | 0.939 | | 140 | | 1.72 | | 1.00 | | 2.96 | 0.050 | |  |
| Frequency child washed their feet | Twice a day | 293 | 1 |  |  |  | 135 | 1 |  |  |  | | 158 | | 1 | |  | |  |  | |  |
|  | Less often | 98 | 1.48 | 0.93 | 2.34 | 0.097 | 30 | 1.69 | 0.75 | 3.80 | 0.202 | | 68 | | 1.08 | | 0.60 | | 1.95 | 0.794 | |  |
| Child’s Soap-use for feet wash | Always | 150 | 1 |  |  |  | 67 | 1 |  |  |  | | 83 | | 1 | |  | |  |  | |  |
|  | Less | 244 | 1.24 | 0.83 | 1.86 | 0.300 | 101 | 1.71 | 0.87 | 3.35 | 0.117 | | 143 | | 0.95 | | 0.55 | | 1.67 | 0.868 | |  |
| Toilet used | Latrine | 325 | 1 |  |  |  | 135 | 1 |  |  |  | | 188 | | 1 | |  | |  |  | |  |
|  | Open defecation | 72 | 1.63 | 0.97 | 2.73 | 0.066 | 33 | 2.16 | 0.99 | 4.68 | 0.052 | | 39 | | 1.46 | | 0.70 | | 3.06 | 0.316 | |  |
| Parental stress score |  |  | 1.03 | 1.01 | 1.06 | 0.013 |  | 1.05 | 1.00 | 1.09 | 0.038 | |  | | 1.03 | | 1.00 | | 1.06 | 0.095 | |  |
| Caregiver depressed | No | 255 | 1 |  |  |  | 110 | 1 |  |  |  | | 143 | | 1 | |  | |  |  | |  |
|  | Yes | 142 | 1.79 | 1.18 | 2.71 | 0.006 | 58 | 1.47 | 0.76 | 2.85 | 0.256 | | 84 | | 2.09 | | 1.17 | | 3.74 | 0.013 | |  |
| Socio-economic status |  |  | 1.36 | 0.67 | 2.79 | 0.397 |  | 2.66 | 0.77 | 9.16 | 0.122 | |  | | 0.63 | | 0.24 | | 1.63 | 0.340 | |  |
| Wall material of child’s sleeping room | Mud | 302 | 1 |  |  |  | 124 | 1 |  |  |  | | 178 | | 1 | |  | |  |  | |  |
|  | Other | 93 | 1.14 | 0.72 | 1.82 | 0.572 | 44 | 1.01 | 0.49 | 2.09 | 0.979 | | 49 | | 1.50 | | 0.76 | | 2.96 | 0.238 | |  |
| Roof material of child’s sleeping room | Natural | 139 | 1 |  |  |  | 67 | 1 |  |  |  | | 72 | | 1 | |  | |  |  | |  |
|  | Iron sheet | 251 | 0.99 | 0.66 | 1.50 | 0.976 | 100 | 1.23 | 0.64 | 2.38 | 0.534 | | 151 | | 0.69 | | 0.38 | | 1.25 | 0.224 | |  |
| Time caregiver spends with index | A lot | 114 | 1 |  |  |  | 55 | 1 |  |  |  | | 59 | | 1 | |  | |  |  | |  |
|  | Not a lot | 273 | 0.59 | 0.38 | 0.92 | 0.021 | 106 | 2.12 | 1.02 | 4.42 | 0.045 | | 167 | | 1.31 | | 0.72 | | 2.40 | 0.380 | |  |
| Caregiver hugged the child | No | 279 | 1 |  |  |  | 110 | 1 |  |  |  | | 167 | | 1 | |  | |  |  | |  |
|  | Yes | 110 | 0.93 | 0.60 | 1.45 | 0.748 | 51 | 0.73 | 0.36 | 1.49 | 0.388 | | 59 | | 1.24 | | 0.67 | | 2.32 | 0.494 | |  |
| More than 1 person share room with child | Yes | 246 | 1 |  |  |  | 106 | 1 |  |  |  | | 140 | | 1 | |  | |  |  | |  |
|  | No | 149 | 0.89 | 0.59 | 1.34 | 0.578 | 62 | 0.70 | 0.36 | 1.38 | 0.306 | | 87 | | 1.62 | | 0.92 | | 2.85 | 0.095 | |  |

^a^ Number of households, ^b^ odds ratio, ^c^ confidence interval, ^d^ p value, ^e^ household head,

## Table S6. Factors associated with household socioeconomic status

Univariable mixed effect linear regression with school ID as random effect

| **Variable** | **categories** | | **N^a^** | **Mean** | **Sd^b^** | **Β^c^** | **95% CI^d^** | | **P^e^** |
| --- | --- | --- | --- | --- | --- | --- | --- | --- | --- |
| Region | Kwale | | 417 | 0.56 | 0.36 | 0 |  |  |  |
|  | Siaya | | 343 | 0.71 | 0.35 | 0.15 | 0.06 | 0.23 | 0.001 |
| Sex of HHH^f^ | Male | | 585 | 0.68 | 0.36 | 0 |  |  |  |
|  | Female | | 175 | 0.47 | 0.34 | -0.22 | -0.27 | -0.16 | <0.000 |
| Number children sleep in main house | 0 | | 187 | 0.74 | 0.34 | 0 |  |  |  |
|  | 1-5 | | 544 | 0.60 | 0.36 | -0.08 | -0.15 | -0.02 | 0.007 |
|  | >5 | | 38 | 0.66 | 0.34 | -0.14 | -0.26 | -0.01 | 0.032 |
| Household tungiasis status | Uninfected | 489 | | 0.63 | 0.36 | 0 |  |  |  |
|  | Infected | 271 | | 0.63 | 0.36 | 0.00 | -0.05 | 0.06 | 0.863 |

^a^ Number of pupils, ^b^ standard deviation, ^c^ β coefficient, ^d^ confidence interval, ^e^ p-value, ^f^ head of household
